# Synthesis and anti-proliferative activity evaluation of *N*3-acyl-*N*5-aryl-3,5-diaminoindazole analogues as anti-head and neck cancer agent

**DOI:** 10.1186/2008-2231-22-4

**Published:** 2014-01-06

**Authors:** Jinho Lee, Jina Kim, Victor Sukbong Hong, Jong-Wook Park

**Affiliations:** 1 Department of Chemistry, Keimyung University, Daegu 704-701, Korea; 2 Department of Immunology, Keimyung University School of Medicine, Daegu 704-701, Korea

**Keywords:** Indazole, 3,5-diaminoindazole, Anticancer, HNSCC

## Abstract

**Background:**

Head and neck squamous cell carcinoma (HNSCC) is the 11th leading cancer by incidence worldwide. Surgery and radiotherapy have been the major treatment for patients with HNSCC while chemotherapy has become an important treatment option for locally advanced HNSCC. Understanding of the molecular mechanisms underlying HNSCC impelled the development of targeted therapeutic agents. The development and combinations of targeted therapies in different cellular pathways may be needed to fulfill the unmet needs of current HNSCC chemotherapy.

**Results:**

A series of *N*3-acyl-*N*5-aryl-3,5-diaminoindazoles were synthesized and their anti-proliferative activities were evaluated against human cancer cell lines, Caki, A549, AMC-HN1, AMC-HN3, AMC-HN4, AMC-HN6, and SNU449. The cellular selectivity of compound was obtained by the modification of substituent at *N*5-aryl group of 3,5-diaminoindazole. Compound 9a and 9b showed more than 7-fold selectivity for AMC-HN4 and AMC-HN3, respectively.

**Conclusions:**

*N*3-acyl-*N*5-aryl-3,5-diaminoindazole analogues can be used as hits in the development of anticancer drug for HNSCC.

## Background

Head and neck squamous cell carcinoma (HNSCC) is the 11th leading cancer by incidence worldwide [1]. The 5-year survival for all stages combined on the basis of Surveillance Epidemiology and End Results (SEER) data is about 60% [2]. The primary risk factors are smoking, smokeless tobacco product, alcohol consumption, and the infection with human papillomavirus (HPV) [3].

Surgery and radiotherapy have been the major treatment for patients with HNSCC. Surgery is a standard treatment but is frequently limited by resectability of tumor and desire for organ preservation. Radiotherapy is used as a single treatment option in early-stage cancers and as an adjuvant treatment. A combination of radiotherapy and chemotherapy has increasingly been used for the treatment of HNSCC. Ten year follow up study of the Head and Neck trials showed that the concomitant non-platinum chemotherapy and radiotherapy reduce recurrences, new tumors, and deaths in patients who have not undergone previous surgery [4]. Chemotherapy has become an important treatment option for locally advanced HNSCC. Bleomycin, taxanes, cisplatin, carboplatin, methotrexate, and 5-fluorouracil (5-FU) are used as chemotherapy regimen in patients with recurrent or metastatic HNSCC and produce response rates from 10% to 40% [2].

Advances in molecular biology increased the knowledge about molecular mechanisms underlying HNSCC and led to the development of targeted therapeutics. Increased EGFR protein expression is observed over 90% of HNSCC. Overexpression of EGFR has been associated with disease recurrence and poor prognosis [5]. Along with the approval of cetuximab (BMS and Merck), monoclonal antibody that blocks the EGFR signaling, clinical trials using small molecular EGFR tyrosine kinase inhibitors have actively been performed. Gefitinib (AstraZeneca) showed a response rate of 10.6% in a phase II study for recurrent/metastatic HNSCC while erlotinib (Roche) demonstrated a response rate of 4.3% in patients with recurrent/metastatic HNSCC. Lapatinib (GSK) in combination with concurrent radiation and cisplatin showed increased complete response rate in phase II/III studies [6]. Lessons learned from clinical studies of EGFR inhibitors suggested the direction for the development of targeted agents for HNSCC. Inhibition of a single growth signaling pathway may not be enough to provide a clinically significant response for HNSCC. Therefore, development and combinations of targeted therapies in different cellular pathways may be needed to fulfill the unmet needs of current HNSCC chemotherapy.

In addition to EGFR overexpression, cyclin D1 overexpression and p53 mutation are frequently occurred in HNSCC. This abnormality may provide cancer cells with limitless replicative potential. Mutations in PI3K-PTEN-AKT signaling pathways are also found in about 10-20% of HNSCC. Activating mutations in PI3K and inactivating mutations of PTEN activate downstream signaling molecules such as Akt/protein kinase B (PKB), mammalian target of rapamycin (mTOR) and ribosomal protein S6 kinase (S6K). It was reported that AKT activation causes reduction of apoptosis as well as increased migration and invasion [7]. Therefore, new therapeutic agents targeting these pathways may provide synergistic effect with clinically advanced EGFR inhibitors when used in combination.

**Figure 1:**
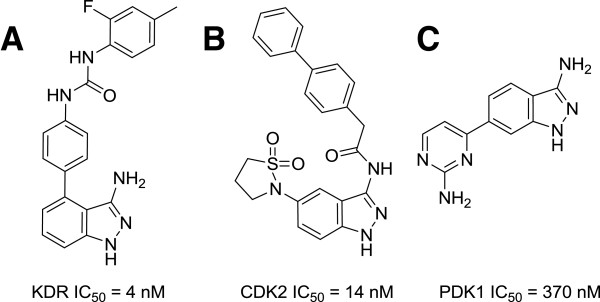
**Structures of kinase inhibitors based on 3-aminoindazole. A**: ABT-869, multitargeted RTK inhibitor such as KDR, cKIT, and FLT3 [9]. **B**: CDK1 and CDK2 inhibitor [8]. **C**: PDK1, Aurora A, CDK2, and IKK1 inhibitor [10].

3-Aminoindazole-based small molecular inhibitors showed strong inhibitory activities against several kinases including CDK1 & 2 [8], KDR, cKIT, FLT3 [9], PDK1 [10] and exhibited potent anti-cancer activity [11]. The structures of representative compound are shown in Figure 1.

Previously, we reported that the treatment of HNSCC cell lines, AMC-HN4 and AMC-HN6, with compound B induced apoptosis in association with growth inhibition, cell cycle arrest, caspase-3 activation, and cytochrome C release [12]. While the compound B showed strong inhibitory effects on cancer cell growth, it had low selectivity, which may pose potential toxicity in *in vivo* studies. As part of our ongoing effort to discover potent and selective kinase inhibitors as potential anticancer agents, a series of 3-aminoindazole derivatives were synthesized and tested for their cancer cell line selectivity.

## Methods

### Chemistry

^1^H- NMR and ^13^C-NMR spectra were recorded on a Bruker AVANEC 400 (400 MHz) spectrometer and chemical shifts (δ) are reported in ppm using tetramethylsilane (TMS) as an internal standard. Mass spectra were obtained using Waters ACQUITY UPLC, Micromass Quattro microTM API. TLC was performed on E. Merck silica gel 60 F254 plates (0.25mm). Silica gel column chromatography was performed using Merck silica gel 60 (230-400 mesh). Unless otherwise noted, all starting materials were obtained from commercially available sources and they were used without further purification. Tetrahydrofuran (THF) was freshly distilled from sodium and benzophenone. All reactions were performed under a nitrogen atmosphere.

#### 5-Bromo-1*H*-indazol-3-ylamine (2)

To a solution of 5-bromo-2-fluorobenzonitrile (3.0 g, 15 mmol) in *n*-butanol (20 mL) was added hydrazine (4.7 mL, 150 mmol). The reaction mixture was refluxed for 6 h. *n*-Butanol was then evaporated, and the residue was dissolved in ethyl acetate. The resulting solution was washed with saturated aqueous Na_2_CO_3_ solution and dried over MgSO_4_. Removal of solvent gave the title compound (2.97 g, 93.4%). ^1^H NMR (DMSO-d_6_, 400 MHz) δ 11.58 (s, 1H, NH_indazole_), 7.92 (s, 1H, H_phenyl_), 7.30 (d, *J* = 8.8 Hz, 1H, H_phenyl_), 7.20 (d, *J* = 8.8 Hz, 1H, H_phenyl_), 5.44 (s, 2H, NH_2__indazole_).

#### N-(5-Bromo-1 *H*-indazol-3-yl)-2-(4-ethoxyphenyl)acetamide (3b)

To a solution of compound **2** (0.80 g, 3.8 mmol) in THF (10 mL) was added 4-ethoxyphenylacetyl chloride (1.9 g, 9.5 mmol). The reaction mixture was refluxed for 10 h under N_2_ atmosphere. After cooled to room temperature, 1N NaOH (14 mL) was added and the reaction mixture was stirred for 2 h. The precipitate formed during evaporation of solvent was collected by filtration and washed with H_2_O. The product was dried in vacuo and obtained 1.2 g in 84.4% yield: ^1^H NMR (DMSO-d_6_, 400 MHz) δ 12.88 (s, 1H, NH_indazole_), 10.70 (s, 1H, NH_amide_), 7.97 (s, 1H, H_phenyl_), 7.42 (s, 2H, H_phenyl_), 7.28 (d, *J* = 8.4 Hz, 2H, H_phenyl_), 6.89 (d, *J* = 8.4 Hz, 2H, H_phenyl_), 4.00 (q, *J* = 6.8 Hz, 2H, CH_2_-O), 3.64 (s, 2H, CH_2_), 1.32 (t, *J* =6.8 Hz, 3H, CH_3_CH_2_-O).

#### N-(5-Bromo-1-trityl-1*H*-indazol-3-yl)-2-(4-ethoxyphenyl)acetamide (4b)

To a solution of compound **3a** (0.070 g, 0.19 mmol) in CH_3_CN (20 mL) were added K_2_CO_3_ (0.040 g, 0.28 mmol) and trityl chloride (0.080 g, 0.28 mmol) and the reaction mixture was refluxed for 12 h. Acetonitrile was then evaporated, and the residue was dissolved in ethyl acetate. The resulting solution was washed with brine and dried over MgSO_4_. The crude product was purified by flash chromatography with a hexane:ethyl acetate (3:1) mixture to provide the title compound (0.060 g, 51%). ^1^H NMR (CDCl_3_, 400 MHz) δ 8.16 (s, 1H, NH_amide_), 7.71 (s, 1H, H_phenyl_), 7.26-7.15 (m, 17H, H_phenyl_), 7.01 (d, *J* = 9.2 Hz, 1H), 6.89 (d, *J* = 8.4 Hz, 2H, H_phenyl_), 6.21 (d, *J* = 9.2 Hz, 1H, H_phenyl_), 4.07 (q, *J* = 6.8 Hz, 2H, CH_2_-O), 3.67 (s, 2H, CH_2_), 1.41 (t, *J* = 6.8 Hz, 3H, CH_3_CH_2_-O).

#### 2-(4-Ethoxyphenyl)-N-[5-(2-fluorophenylamino)-1-trityl-1*H*-indazol-3-yl]acetamide (5b)

To a solution of compound **4a** (0.040 g, 0.065 mmol) in toluene (2 mL) were added 2-fluoroaniline (0.008 mL, 0.08 mmol), sodium *tert*-butoxide (0.013 g, 0.14 mmol), Pd_2_(dba)_3_ (0.001 g, 0.001 mmol), and (R)-BINAP (0.0015 g, 0.0023 mmol). The reaction mixture was refluxed for 4 h under N_2_ atmosphere. Solvents were evaporated, and the residue was treated with ethyl acetate. The resulting mixture was washed with brine and dried over MgSO_4_. The crude product was purified by flash chromatography with a hexane:ethyl acetate (3:1) mixture to provide the title compound (0.016 g, 38%). ^1^H NMR (CDCl_3_, 400 MHz) δ 7.93 (d, *J* = 7.6 Hz, 1H, H_phenyl_), 7.82 (s, 1H, H_phenyl_), 7.23-7.20 (m, 19H, H_phenyl_), 7.00-6.94 (m, 2H, H_phenyl_), 6.87 (d, *J* = 6.8 Hz, 2H, H_phenyl_), 6.36 (d, *J* = 8.4 Hz, 1H, H_phenyl_), 4.01 (q, *J* = 6.8 Hz, 2H, CH_2_-O), 3.66 (s, 2H, CH_2_), 1.42 (t, *J* = 6.8 Hz, 3H, CH_3_CH_2_-O).

#### 2-(4-Ethoxyphenyl)-N-[5-(2-fluorophenylamino)-1*H*-indazol-3-yl]acetamide (6b)

To a solution of compound **5a** (0.040 g, 0.062 mmol) in CH_2_Cl_2_ (5 mL) were added trifluoroacetic acid (0.16 mL), phenol (0.013 mL), water (0.014 mL), and triisopropylsilane (0.007 mL) and the reaction mixture was stirred for 4 h at room temperature. Solvents were then evaporated, and the residue was dissolved in ethyl acetate. The resulting solution was washed with saturated aqueous Na_2_CO_3_ solution and dried over MgSO_4_. The crude product was purified by flash chromatography with a dichloromethane:methanol (95:5) mixture to provide the title compound (0.01 g, 40 %). ^1^H NMR (CDCl_3_, 400 MHz) δ 7.77 (s, 1H, H_phenyl_), 7.66 (s, 1H, H_phenyl_), 7.28-7.23 (m, 2H, H_phenyl_), 7.13-7.04 (m, 2H, H_phenyl_), 6.97 (t, *J* = 7.2 Hz, 1H, H_phenyl_), 6.90 (d, *J* = 7.6 Hz, 2H, H_phenyl_), 6.77 (m, 1H, H_phenyl_), 5.87 (s, 1H, H_phenyl_), 4.02 (q, *J* = 6.8 Hz, 2H, CH_2_-O), 3.75 (s, 2H, CH_2_), 1.42 (t, *J* = 6.8 Hz, 3H, CH_3_CH_2_-O); ^13^C NMR (CDCl_3_, 100 MHz) δ 169.9 (CO), 158.6 (C_phenyl_), 151.0 (C_phenyl_), 140.3 (C_phenyl_), 138.8 (C_phenyl_), 134.8 (C_phenyl_), 130.7 (CH_phenyl_), 127.6 (C_phenyl_), 126.0 (C_phenyl_), 124.4 (CH_phenyl_), 124.2 (CH_phenyl_), 119.2 (CH_phenyl_), 119.1 (CH_phenyl_), 116.9 (C_phenyl_), 115.3 (CH_phenyl_), 115.1 (CH_phenyl_), 113.3 (CH_phenyl_), 110.6 (CH_phenyl_), 63.5 (CH_2_-O), 43.2 (CH_2_), 14.8 (CH_3_).

#### 2-(1,1′-Biphenyl-4-yl)-N-(5-(2-fluorophenylamino)-1*H*-indazol-3-yl)acetamide (6a)

The title compound was synthesized using the same procedure used for the synthesis of **6b**.

^1^H NMR (DMSO-d_6_, 400 MHz) δ 12.17(s, 1H, NH_indazole_), 10.58 (s, 1H, NH_amide_), 8.10 (s, 1H, H_phenyl_), 7.66-7.59 (m, 4H, H_phenyl_), 7.47-7.43 (m, 4H, H_phenyl_), 7.36-7.32 (m, 2H, H_phenyl_), 7.25-7.20 (m, 1H, H_phenyl_), 7.11 (t, *J* = 7.6 Hz, 1H, H_phenyl_), 6.99-6.94 (m, 1H, H_phenyl_), 6.79-6.76 (m, 2H, H_phenyl_), 3.74 (s, 1H, CH_2_); ^13^C NMR (DMSO-d_6_, 100 MHz): δ 169.4 (CO), 169.3 (C_phenyl_), 155.7 (C_phenyl_), 153.3 (C_phenyl_), 143.2 (C_phenyl_), 143.1 (C_phenyl_), 140.8 (C_phenyl_), 140.6 (CH_phenyl_), 138.9 (CH_phenyl_), 136.0 (CH_phenyl_), 131.2 (C_phenyl_), 130.2 (CH_phenyl_), 129.4 (CH_phenyl_), 127.8 (CH_phenyl_), 127.1 (CH_phenyl_), 125.3 (CH_phenyl_), 123.5 (CH_phenyl_), 122.7 (C_phenyl_), 121.3 (CH_phenyl_), 116.6 (CH_phenyl_), 113.3 (CH_phenyl_), 111.0 (C_phenyl_), 93.8 (C_phenyl_), 42.4 (CH_2_).

General procedure for the synthesis of compound **9a** to **9h**.

#### 5-Nitro-1*H*-indazol-3-ylamine

To a solution of 2-fluoro-5-nitrobenzonitrile (5 g, 30.1 mmol) in *n*-butanol (20 mL) was added hydrazine (2.8 mL, 90 mmol). The reaction mixture was refluxed for 4 h, and *n*-butanol was evaporated. The precipitate formed during evaporation was collected by filtration and washed with H_2_O. The product was dried in vacuo and obtained 5.0 g in 93.2 % yield. ^1^H NMR (DMSO-d_6_, 400 MHz) δ 12.18 (s, 1H, NH_indazole_), 8.90 (s, 1H, H_phenyl_), 8.05 (d, *J* = 9.0 Hz, 1H, H_phenyl_), 7.34 (d, *J* = 9.0 Hz, 1H, H_phenyl_), 6.01 (s, 2H, NH_2 indazole_).

#### 2-(4-Ethoxyphenyl)-N-(5-nitro-1*H*-indazol-3-yl)acetamide

To a solution of 5-nitro-1H-indazol-3-ylamine (5.0 g, 28 mmol) in THF (20 mL) was added 4-ethoxyphenylacetyl chloride (11.1 g, 56 mmol). The reaction mixture was refluxed for 5 h under N_2_ atmosphere. After cooled to room temperature, 2N NaOH (40 mL) was added and the reaction mixture was stirred for 2 h. The precipitate formed during evaporation was collected by filtration and washed with H_2_O. The product was dried in vacuo and obtained 7.2 g in 75.6% yield. ^1^H NMR (DMSO-d_6_, 400 MHz) δ 11.05 (s, 1H, NH_indazole_), 9.00 (d, *J* = 2.2 Hz, 1H, H_phenyl_), 8.14 (dd, *J* = 2.2, 9.2 Hz, 1H, H_phenyl_), 7.60 (d, *J* = 9.2 Hz, 1H, H_phenyl_), 7.29 (d, *J* = 8.4 Hz, 2H, H_phenyl_), 6.89 (d, *J* = 8.4 Hz, 2H, H_phenyl_), 3.99 (q, *J* = 6.8 Hz, 2H, CH_2_-O), 3.70 (s, 2H, CH_2_), 1.31 (t, *J* = 6.8 Hz, 3H, CH_3_CH_2_-O).

#### 2-(4-Ethoxyphenyl)-N-(5-nitro-1-trityl-1*H*-indazol-3-yl)acetamide

To a solution of 2-(4-ethoxyphenyl)-N-(5-nitro-1H-indazol-3-yl)acetamide (7.2 g, 21 mmol) in CH_3_CN (100 mL) were added triethylamine (8.8 mL, 63 mmol) and trityl chloride (8.8 g, 32 mmol). The resulting mixture was heated to reflux for 3 h. The precipitate formed during evaporation of solvent was collected. The crude product was purified by recrystallization using a mixture of dichloromethane and hexane to provide the title compound (9.18 g, 75%). ^1^H NMR (CDCl_3_, 400 MHz) δ 7.90 (s, 1H, H_phenyl_), 7.80 (dd, *J* = 2, 9.5 Hz, 1H, H_phenyl_), 7.31-7.13 (m, 17H, H_phenyl_), 6.90 (d, *J* = 8 Hz, 2H, H_phenyl_), 6.37 (d, *J* = 9.5 Hz, 1H, H_phenyl_), 4.03 (q, *J* = 6.8 Hz, 2H, CH_2_-O), 3.73 (s, 2H, CH_2_), 1.26 (t, *J* = 6.8 Hz, 3H, CH_3_CH_2_-O).

#### N-(5-Amino-1-trityl-1*H*-indazol-3-yl)-2-(4-ethoxyphenyl)acetamide (7)

To a solution of 2-(4-Ethoxyphenyl)-N-(5-nitro-1-trityl-1H-indazol-3-yl)acetamide (3.45 g, 5.9 mmol) in methanol:dichloromethane (7:1) mixture was added catalytic amount of Pd/C. The resulting mixture was stirred for 12 h under H_2_ atmosphere. The reaction mixture was filtered through a plug of celite and purified by flash chromatography with hexane:ethyl acetate (1:1) mixture to provide the title compound (2.2 g, 67.5%). ^1^H NMR (CDCl_3_, 400 MHz) δ 7.25-7.18 (m, 17H, H_phenyl_), 7.09 (d, *J* = 1.8 Hz, 1H, H_phenyl_), 6.87 (d, *J* = 8.8 Hz, 2H, H_phenyl_), 6.42 (dd, *J* = 1.8, 9.1 Hz, 1H, H_phenyl_), 6.15 (d, *J* = 9.1 Hz, 1H, H_phenyl_), 4.02 (q, *J* = 7.0 Hz, 2H, CH_2_-O), 3.65 (s, 2H, CH_2_), 1.41 (t, *J* = 7.0 Hz, 3H, CH_3_CH_2_-O).

#### 4-{3-[2-(4-Ethoxyphenyl)acetylamino]-1-trityl-1*H*-indazol-5-ylamino}-3-fluorobenzoic acid ethyl ester

To a solution of compound **7** (2.0 g, 3.6 mmol) in toluene (10 mL) were added ethyl 4-bromo-3-fluorobenzoate (1.34 g, 5.4 mmol), sodium *tert*-butoxide (0.70 g, 7.2 mmol), Pd_2_(dba)_3_ (0.10 g, 0.11 mmol), and (R)-BINAP (0.10 g, 0.16 mmol). The reaction mixture was refluxed for 6 h under N_2_ atmosphere. Solvents were evaporated, and the crude product was purified by flash chromatography with a hexane:ethyl acetate (3:1) mixture to provide the title compound (1.57 g, 60.3%). ^1^H NMR (CDCl_3_, 400 MHz): δ 7.80 (s, 1H, H_phenyl_), 7.68-7.63 (m, 2H, H_phenyl_), 7.17-7.15 (m, 15H, H_phenyl_), 7.02 (d, *J* = 8.2 Hz, 2H, H_phenyl_), 6.83 (d, *J* = 9.0 Hz, 1H, H_phenyl_), 6.70 (d, *J* = 8.2 Hz, 2H, H_phenyl_), 6.36 (d, *J* = 9.0 Hz, 1H, H_phenyl_), 6.31 (s, 1H, H_phenyl_), 4.27 (q, *J* = 6.8 Hz, 2H, CH_2_-OCO), 3.83 (q, *J* = 6.8 Hz, 2H, CH_2_-O), 3.31 (s, 2H, CH_2_), 1.31 (t, *J* = 6.8 Hz, 6H, CH_3_CH_2_-O).

#### 4-{3-[2-(4-Ethoxyphenyl)acetylamido]-1-trityl-1*H*-indazol-5-ylamino}-3-fluorobenzoic acid (8)

To a solution of 4-{3-[2-(4-Ethoxyphenyl)acetylamido]-1-trityl-1H-indazol-5-ylamino}-3-fluorobenzoic acid ethyl ester (1.3 g, 1.8 mmol) in THF:methanol:H_2_O (3:1:1) mixture was added LiOH · H_2_O (0.4 g, 10 mmol). The resulting mixture was refluxed for 2 hr. Solvents were evaporated and the crude product was purified by flash chromatography with hexane:ethyl acetate(1:2) mixture to provide the title compound (0.89 g, 71.6%). ^1^H NMR (CDCl_3_, 400 MHz) δ 7.74 (s, 1H, H_phenyl_), 7.68-7.65 (m, 2H, H_phenyl_), 7.24-7.20 (m, 17H, H_phenyl_), 7.04 (t, *J* = 8.4 Hz, 1H, H_phenyl_), 6.89 (dd, *J* = 1.6, 9.2 Hz, 1H, H_phenyl_), 6.83 (d, *J* = 8.4 Hz, 2H, H_phenyl_), 6.38 (d, *J* = 9.2 Hz, 1H, H_phenyl_), 3.97 (q, *J* = 6.8 Hz, 2H, CH_2_-O), 3.59 (s, 2H, CH_2_), 1.37 (t, *J* = 6.8 Hz, 3H, CH_3_CH_2_-O).

#### 2-(4-Ethoxyphenyl)-N-{5-[2-fluoro-4-(morpholine-4-carbonyl)phenylamino]-1-trityl-1*H*-indazol-3-yl}acetamide

To a solution of compound **8** (0.1 g, 0.15 mmol) in DMF (10 mL) were added morpholine (0.015 mL, 0.17 mmol), EDC (0.058 g, 0.3 mmol), and HOBt (0.041 g, 0.3 mmol). The resulting solution was stirred for 9 h at room temperature. Solvents were then evaporated, and the residue was dissolved in ethyl acetate. The resulting solution was washed with saturated aqueous Na_2_CO_3_ solution and dried over MgSO_4_. The crude product was purified by flash chromatography with a dichloromethane:methanol (95:5) mixture to provide the title compound (0.10 g, 88 %). ^1^H NMR (CDCl_3_, 400 MHz): δ 8.35 (s, 1H, H_phenyl_), 7.73 (s, 1H, H_phenyl_), 7.20-7.17 (m, 15H, H_phenyl_), 7.12 (d, J = 8.8 Hz, 2H, H_phenyl_), 7.05-7.01 (m, 1H, H_phenyl_), 6.83 (dd, J = 2.4, 9.2 Hz, 1H, H_phenyl_), 6.78 (d, J = 8.4 Hz, 2H, H_phenyl_), 6.32 (d, J = 9.2 Hz, 1H, H_phenyl_), 6.06 (d, J = 2.4 Hz, 1H, H_phenyl_), 3.93 (q, J = 7.2 Hz, 2H, CH_2_-O), 3.65-3.60 (m, 8H, CH_2 morpholine_), 3.47 (s, 2H, CH_2_), 1.36 (t, J = 6.8 Hz, 3H, CH_3_CH_2_-O).

#### 2-(4-Ethoxyphenyl)-N-{5-[2-fluoro-4-(morpholine-4-carbonyl)phenylamino]-1*H*-indazol-3-yl}acetamide (9a)

Trityl protecting group was removed using the method which was used for the synthesis of compound **6b**. The product was obtained in 73% yield (0.050 g). ^1^H NMR (CDCl_3_ + CD_3_OD, 400 MHz) δ 7.49 (s, 1H, H_phenyl_), 7.41 (d, *J* = 8.8 Hz, 1H, H_phenyl_), 7.27 (d, *J* = 8.8 Hz, 2H, H_phenyl_), 7.25-7.18 (m, 2H, H_phenyl_), 7.11 (t, *J* = 8.4 Hz, 1H, H_phenyl_), 7.04 (d, *J* = 8.4 Hz, 1H, H_phenyl_), 6.82 (d, *J* = 8.4 Hz, aromatic, 2H, H_phenyl_), 3.96 (q, *J* = 6.8 Hz, 2H, CH_2_-O), 3.64 (m, 10H, CH_2_, CH_2 morpholine_), 1.34 (t, *J* = 6.8 Hz, 3H, CH_3_CH_2_-O); ^13^C NMR (CDCl_3_ + CD_3_OD, 100 MHz) δ 172.1 (CONH), 170.4 (COmorpholine), 158.1 (C_phenyl_), 152.3 (C_phenyl_), 149.9 (C_phenyl_), 139.3 (C_phenyl_), 138.7 (C_phenyl_), 136.5 (C_phenyl_), 133.8 (C_phenyl_), 129.8 (CH_phenyl_), 127.1 (CH_phenyl_), 124.1 (CH_phenyl_), 123.7 (C_phenyl_), 116.9 (C_phenyl_), 114.6 (CH_phenyl_), 114.3 (CH_phenyl_), 113.3 (CH_phenyl_), 112.3 (CH_phenyl_), 110.9 (CH_phenyl_), 66.4 (O-CH_2 morpholine_), 63.1 (CH_2_-O), 48.5 (CH_2_), 41.6 (N-CH_2 morpholine_), 13.8 (CH_3_); ESI MS: m/z = 518 [M + H] ^+^.

#### 2-(4-Ethoxyphenyl)-N-{5-[2-fluoro-4-(4-methylpiperazine-1-carbonyl)phenylamino]-1*H*-indazol-3-yl}acetamide (9b)

^1^H NMR (CDCl_3_ + CD_3_OD, 400 MHz) δ 7.49 (s, 1H, H_phenyl_), 7.42 (d, *J* = 8.8 Hz, 1H, H_phenyl_), 7.28 (d, *J* = 8.6 Hz, 2H, H_phenyl_), 7.25 (dd, *J* = 1.6, 8.4 Hz, 1H, H_phenyl_), 7.19 (dd, *J* = 1.2, 8.8 Hz, 1H, H_phenyl_), 7.12 (t, *J* = 8.4 Hz, 1H, H_phenyl_), 7.05 (dd, *J* = 1.6, 8.4 Hz, 1H, H_phenyl_), 6.84 (d, *J* = 8.6 Hz, 2H, H_phenyl_), 3.98 (q, *J* = 7.2 Hz, 2H, CH_2_-O), 3.67-3.65 (m, 6H, CH_2_), 2.45 (s, 4H, CH_2_), 2.31 (s, 3H, CH_3_-N), 1.35 (t, *J* = 7.2 Hz, 3H, CH_3_CH_2_-O); ^13^C NMR (CDCl_3_ + CD_3_OD, 100 MHz) δ 172.1 (CONH), 170.3 (COmorpholine), 158.1 (C_phenyl_), 152.3 (C_phenyl_), 149.9 (C_phenyl_), 139.3 (C_phenyl_), 138.7 (C_phenyl_), 136.5 (C_phenyl_), 133.9 (C_phenyl_), 130.3 (CH_phenyl_), 129.3 (CH_phenyl_), 127.2 (CH_phenyl_), 124.6 (CH_phenyl_), 123.8 (CH_phenyl_), 116.9 (CH_phenyl_), 114.4 (CH_phenyl_), 112.8 (CH_phenyl_), 111.6 (C_phenyl_), 110.31 (C_phenyl_), 63.1 (CH_2_-O), 54.4 (CH_2_-N), 48.8 (CH_2_-N), 44.6 (CH_3_-N), 41.6 (CH_2_), 13.8 (CH_3_); ESI MS: m/z = 531 [M + H]^+^.

#### N-(2-Dimethylaminoethyl)-4-{3-[2-(4-ethoxyphenyl)acetylamido]-1*H*-indazol-5-ylamino}-3-fluorobenzamide (9c)

^1^H NMR(CD_3_OD, 400 MHz) δ 7.58 (d, *J* = 12.8 Hz, 1H, H_phenyl_), 7.50 (s, 1H, H_phenyl_), 7.47 (d, *J* = 10 Hz, 1H, H_phenyl_), 7.43 (d, *J* = 8.8 Hz, 1H, H_phenyl_), 7.29 (d, *J* = 8.6 Hz, 2H, H_phenyl_), 7.26 (d, *J* = 10 Hz, 1H, H_phenyl_), 7.08 (t, *J* = 8.4 Hz, 1H, H_phenyl_), 6.83 (d, *J* = 8.6 Hz, 2H, H_phenyl_), 3.95 (q, *J* = 6.8 Hz, 2H, CH_2_-O), 3.68 (s, methylenic, 2H, CH_2_), 3.50 (t, *J* = 6.8 Hz, 2H, CH_2_-N), 2.59 (t, *J* = 6.8 Hz, 2H, CH_2_-N), 2,33 (s, 6H, CH_3_-N), 1.34 (t, *J* = 6.8 Hz, 3H, CH_3_CH_2_-O); ^13^C NMR(CD_3_OD, 100 MHz) δ 172.2 (CONH), 167.5 (CONH), 158.1 (C_phenyl_), 152.3 (C_phenyl_), 150.0 (C_phenyl_), 139.3 (C_phenyl_), 138.8 (C_phenyl_), 137.6 (C_phenyl_), 133.7 (C_phenyl_), 130.3 (CH_phenyl_), 129.3 (CH_phenyl_), 127.1 (CH_phenyl_), 124.2 (C_phenyl_), 123.2 (CH_phenyl_), 116.9 (CH_phenyl_), 114.3 (CH_phenyl_), 113.2 (CH_phenyl_), 111.4 (CH_phenyl_), 110.4 (C_phenyl_), 63.0 (CH_2_-O), 57.9 (CH_2_-N), 44.0 (CH_2_-N), 41.6 (CH_2_), 37.0 (CH_3_-N), 13.8 (CH_3_); ESI MS: m/z = 519 [M + H]^+^.

#### N-(2-Diethylaminoethyl)-4-{3-[2-(4-ethoxyphenyl)acetylamido]-1*H*-indazol-5-ylamino}-3-fluorobenzamide (9d)

^1^H NMR (CD_3_OD, 400 MHz) δ 7.57 (d, *J* = 12.8 Hz, 1H, H_phenyl_), 7.50 (s, 1H, H_phenyl_), 7.47 (d, *J* = 9.2 Hz, 1H, H_phenyl_), 7.44 (d, *J* = 8.8 Hz, 1H, H_phenyl_), 7.31-7.25 (m, 3H, H_phenyl_), 7.10 (t, *J* = 8.8 Hz, 1H, H_phenyl_), 6.84 (d, *J* = 8.4 Hz, 2H, H_phenyl_), 3.97 (q, *J* = 6.8 Hz, 2H, CH_2_-O), 3.69 (s, 2H, CH_2_), 3.49 (t, *J* = 7.2 Hz, 2H, CH_2_-NCO), 2.75-2.66 (m, 6H, CH_2_-N), 1.35 (t, *J* = 6.8 Hz, 3H, CH_3_CH_2_-O), 1.11 (t, *J* = 7.2 Hz, 6H, CH_3_CH_2_-N); ^13^C NMR (CD_3_OD, 100 MHz) δ 172.2 (CONH), 167.5 (CONH), 158.1 (C_phenyl_), 152.3 (C_phenyl_), 149.9 (C_phenyl_), 139.3 (C_phenyl_), 138.8 (C_phenyl_), 137.6 (C_phenyl_), 133.6 (C_phenyl_), 130.3 (CH_phenyl_), 129.3 (CH_phenyl_), 127.1 (CH_phenyl_), 124.2 (C_phenyl_), 123.1 (CH_phenyl_), 116.9 (CH_phenyl_), 114.7 (CH_phenyl_), 113.3 (CH_phenyl_), 110.4 (CH_phenyl_), 110.3 (C_phenyl_), 63.0 (CH_2_-O), 51.3 (CH_2_-N), 46.2 (CH_2_-N), 41.6 (CH_2_), 36.7 (CH_2_-N), 13.8 (CH_3_), 10.0 (CH_3_); ESI MS: m/z = 547 [M + H]^+^.

#### 4-{3-[2-(4-Ethoxyphenyl)acetylamido]-1*H*-indazol-5-ylamino}-3-fluoro-N-(2-morpholin-4-yl-ethyl)benzamide (9e)

^1^H NMR (CD_3_OD, 400 MHz) δ 7.56 (d, *J* = 12.4 Hz, 1H, H_phenyl_), 7.51 (s, 1H, H_phenyl_), 7.44 (d, *J* = 8.8 Hz, 1H, H_phenyl_), 7.40 (d, *J* = 9.2 Hz, 1H, H_phenyl_), 7.27-7.22 (m, 3H, H_phenyl_), 7.07 (t, *J* = 8.4 Hz, 1H, H_phenyl_), 6.79 (d, *J* = 8.4 Hz, 2H, H_phenyl_), 3.91 (q, *J* = 6.8 Hz, 2H, CH_2_-O), 3.68-3.66 (m, 6H, CH_2_), 3.48 (t, *J* = 6.8 Hz, 2H, CH_2_), 2.54 (t, *J* = 6.8 Hz, 2H, CH_2_), 2.49 (s, 4H, CH_2_), 1.31 (t, *J* = 6.8 Hz, 3H, CH_3_CH_2_-O); ^13^C NMR (CD_3_OD, 100 MHz) δ 172.2 (CONH), 167.5 (CONH), 158.1 (C_phenyl_), 152.3 (C_phenyl_), 149.9 (C_phenyl_), 139.6 (C_phenyl_), 137.6 (C_phenyl_), 133.6 (CH_phenyl_), 129.8 (CH_phenyl_), 128.2 (C_phenyl_), 127.1 (CH_phenyl_), 124.2 (CH_phenyl_), 123.7 (CH_phenyl_), 123.2 (C_phenyl_), 116.9 (C_phenyl_), 114.3 (CH_phenyl_), 113.4 (C_phenyl_), 112.7 (CH_phenyl_), 110.9 (CH_phenyl_), 67.3 (CH_2_-O_morpholine_), 63.0 (CH_2_-O), 57.4 (CH_2_-N_morpholine_), 53.3 (CH_2_-N), 41.6 (CH_2_), 36.23 (CH_2_-NCO), 13.8(CH_3_); ESI MS: m/z = 561 [M + H]^+^.

#### N-(3-Dimethylaminopropyl)-4-{3-[2-(4-ethoxyphenyl)acetylamido]-1*H*-indazol-5-ylamino}-3-fluorobenzamide (9f)

^1^H NMR (CD_3_OD, 400 MHz) δ 7.56 (dd, *J* = 2.0, 12.6 Hz, 1H, H_phenyl_), 7.55 (d, *J* = 1.6 Hz, 1H, H_phenyl_), 7.45 (dd, *J* = 1.6, 8.4 Hz, 1H, H_phenyl_), 7.41 (d, *J* = 9.2 Hz, 1H, H_phenyl_), 7.27 (d, *J* = 8.4 Hz, 2H, H_phenyl_), 7.26-7.23 (m, 1H, H_phenyl_), 7.08 (t, *J* = 8.8 Hz, 1H, H_phenyl_), 6.81 (d, *J* = 8.8 Hz, 2H, H_phenyl_), 3.93 (q, *J* = 7.2 Hz, 2H, CH_2_-O), 3.67 (s, 2H, CH_2_), 3.36 (t, *J* = 6.8 Hz, 2H, CH_2_), 2.44 (t, *J* = 7.2 Hz, 2H, CH_2_), 2.28 (s, 6H, CH_3_-N), 1.78 (m, 2H, CH_2_), 1.33 (t, *J* = 7.2 Hz, 3H, CH_3_CH_2_-O); ^13^C NMR (CD_3_OD, 100 MHz) δ 172.2 (CONH), 167.4 (CONH), 158.1 (C_phenyl_), 152.3 (C_phenyl_), 149.9 (C_phenyl_), 139.3 (C_phenyl_), 138.8 (C_phenyl_), 137.6 (C_phenyl_), 133.6 (C_phenyl_), 130.3 (CH_phenyl_), 129.3 (CH_phenyl_), 127.1 (CH_phenyl_), 124.2 (C_phenyl_), 123.3 (CH_phenyl_), 116.9 (CH_phenyl_), 114.3 (CH_phenyl_), 113.3 (CH_phenyl_), 111.4 (CH_phenyl_), 110.4 (C_phenyl_), 63.0 (CH_2_-O), 56.8 (CH_2_), 43.9 (CH_2_), 41.6 (CH_2_), 37.7 (CH_2_), 26.7 (CH_3_), 13.8 (CH_3_); ESI MS: m/z = 533 [M + H]^+^.

#### N-(3-Diethylaminopropyl)-4-{3-[2-(4-ethoxyphenyl)acetylamido]-1*H*-indazol-5-ylamino}-3-fluorobenzamide (9g)

^1^H NMR (CD_3_OD, 400 MHz) δ 7.56 (dd, *J* = 2.0, 12.6 Hz, 1H, H_phenyl_), 7.50 (s, 1H, H_phenyl_), 7.45 (d, *J* = 8.4 Hz, 1H, H_phenyl_), 7.42 (d, *J* = 9.2 Hz, 1H, H_phenyl_), 7.28-7.23 (m, 3H, H_phenyl_), 7.09 (t, *J* = 8.4 Hz, 1H, H_phenyl_), 6.81 (d, *J* = 8.8 Hz, 2H, H_phenyl_), 3.94 (q, *J* = 7.2 Hz, 2H, CH_2_-O), 3.67 (s, 2H, CH_2_), 3.37-3.35 (m, 2H, CH_2_), 2.61-2.55 (m, 6H, CH_2_), 1.77 (m, 2H, CH_2_), 1.33 (t, *J* = 7.2 Hz, 3H, CH_3_CH_2_-O), 1.04 (t, *J* = 7.2 Hz, 6H, CH_3_); ^13^C NMR (CD_3_OD, 100 MHz) δ 172.2 (CONH), 167.4 (CONH), 158.1 (C_phenyl_), 152.3 (C_phenyl_), 149.9 (C_phenyl_), 139.3 (C_phenyl_), 138.8 (C_phenyl_), 137.6 (C_phenyl_), 133.6 (C_phenyl_), 130.3 (CH_phenyl_), 129.3 (CH_phenyl_), 127.1 (CH_phenyl_), 124.2 (C_phenyl_), 123.3 (CH_phenyl_), 116.9 (CH_phenyl_), 114.5 (CH_phenyl_), 113.2 (CH_phenyl_), 111.4 (CH_phenyl_), 110.3 (C_phenyl_), 63.0 (CH_2_-O), 50.0 (CH_2_), 46.3 (CH_2_), 41.6 (CH_2_), 38.1 (CH_2_), 25.5 (CH_2_), 13.3 (CH_3_), 9.8(CH_3_); ESI MS: m/z = 561 [M + H]^+^.

#### N-(3-Dimethylaminopropyl)-4-{3-[2-(4-ethoxyphenyl)acetylamido]-1*H*-indazol-5-ylamino}-3-fluoro-N-methylbenzamide (9h)

^1^H NMR (CD_3_OD, 400 MHz) δ 7.49 (s, 1H, H_phenyl_), 7.43 (d, *J* = 8.8 Hz, 1H, H_phenyl_), 7.29 (d, *J* = 8.4 Hz, 2H, H_phenyl_), 7.26 (d, *J* = 10.4 Hz, 1H, H_phenyl_), 7.19 (d, *J* = 12.4 Hz, 1H, H_phenyl_), 7.13 (t, *J* = 8.4 Hz, 1H, H_phenyl_), 7.07 (m, 1H, H_phenyl_), 6.85 (d, *J* = 8.4 Hz, 2H, H_phenyl_), 3.99 (q, *J* = 7.0 Hz, 2H, CH_2_-O), 3.68(s, 2H, CH_2_), 3.06 (s, 3H, CH_3_), 2.30-2.15 (m, 8H, CH_2_, CH_3_), 1.83 (m, 2H, CH_2_), 1.35 (t, *J* = 7.0 Hz, 3H, CH_3_CH_2_-O), 1.28 (s, 2H, CH_2_); ^13^C NMR (CD_3_OD, 100 MHz) δ 170.0 (CONH), 157.7 (CONH), 152.3 (C_phenyl_), 149.9 (C_phenyl_), 140.1 (C_phenyl_), 138.6 (C_phenyl_), 135.5 (C_phenyl_), 133.8 (CH_phenyl_), 131.0 (CH_phenyl_), 130.0 (CH_phenyl_), 128.3 (CH_phenyl_), 126.5 (C_phenyl_), 124.1 (C_phenyl_), 117.1 (CH_phenyl_), 115.1 (CH_phenyl_), 114.2 (CH_phenyl_), 113.4 (C_phenyl_), 111.8 (CH_phenyl_), 110.8 (C_phenyl_), 63.4 (CH_2_-O), 56.7 (CH_2_-N), 46.3 (CH_2_-NCO), 45.7 (CH_3_-N), 45.2 (CH_2_), 41.8 (CH_3_-NCO), 15.5 (CH_2_), 14.8 (CH_3_); ESI MS: m/z = 547 [M + H]^+^.

### Biological assay

### Cell growth inhibition assay (SRB assay)

The sulforhodamine B (SRB) assay was carried out as previously described [13]. Briefly, the cells were plated in 96-well culture plates at a density of 3,000 cells/well in phenol red free-medium and allowed to attach for 10 h. After 24 h or 48 h treatment of compounds, culture media were removed. 0.07 mL of 0.4% (w/v) SRB (Sigma) in 1% acetic acid solution were added to each well and left at room temperature for 20 min. SRB was removed and the plates washed 5 times with 1% acetic acid before air drying. Bound SRB was solubilized with 0.2 mL of 10 mM unbuffered Tris-base solution (Sigma) and plates were left on a plate shaker for at least 10 min. Absorbance was read in a 96-well plate reader at 492 nm subtracting the background measurement at 620 nm. The test optical density (OD) value was defined as the absorbance of each individual well, minus the blank value (‘blank’ is the mean OD of the background control wells).

## Results and discussion

**Figure 2:**
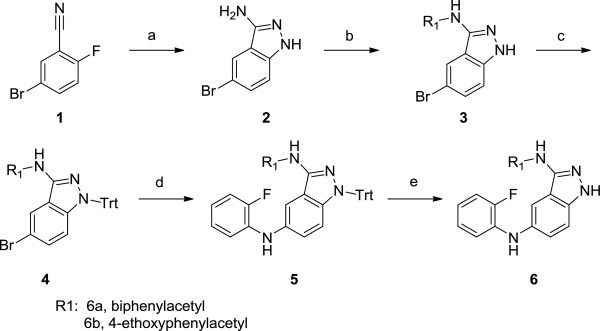
**Synthetic scheme for compound 6a and 6b.** Reagents and experimental conditions: **a)** H_2_NNH_2_, *n*-BuOH, reflux, **b)** i) 4*-*C_2_H_5_OC_6_H_4_CH_2_COCl (or 4*-*PhC_6_H_4_CH_2_COCl), THF, reflux, ii) 2N NaOH, **c)** TrtCl, K_2_CO_3_, CH_3_CN, reflux, **d)** 2*-*FC_6_H_4_NH_2_, Pd_2_(dba)_3_, (R)-BINAP, NaOBu*-t*, toluene, reflux, **e)** TFA:Phenol:H_2_O:TIPS (88:5:5:2), DCM.

**Figure 3:**
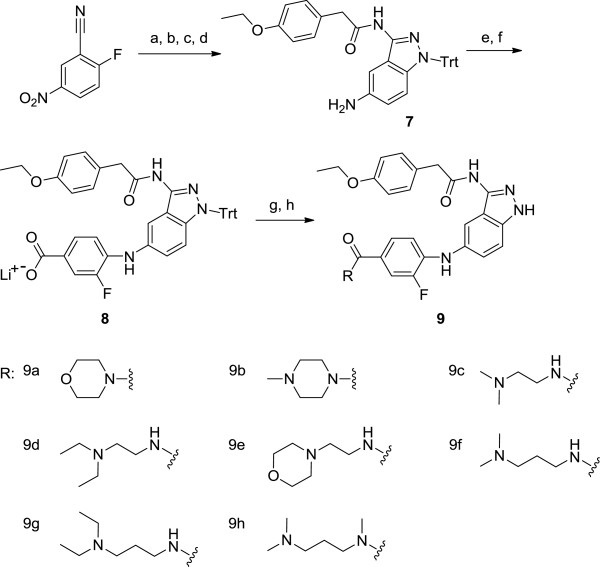
**Synthetic scheme for compound 9a to 9h.** Reagents and experimental conditions: **a)** H_2_NNH_2_, *n*-BuOH, reflux, **b)** i) *p-*C_2_H_5_OC_6_H_4_CH_2_COCl, THF, reflux, ii) 2N NaOH **c)** TrtCl, Et_3_N, CH_3_CN, reflux, **d)** Pd/C, H_2_, MeOH/DCM, **e)** 2-F-4-EtO_2_CC_6_H_3_Br, Pd_2_(dba)_3_, (R)-BINAP, NaOBu*-t*, toluene, reflux, **f)** LiOH, THF:H_2_O:MeOH (3:1:1), reflux, **g)** RH, EDC, HOBT, DMF, **h)** TFA, DCM.

*N*3-Acyl-*N*5-aryl-3,5-diaminoindazole derivatives were synthesized using two different procedures (Figures 2 and 3). Figure 2 was used to synthesize compound **6a** and **6b** which had no additional substitution at 2-fluoroaniline ring. 3-Amino-5-bromoindazole was synthesized from 5-bromo-2-fluoronitrile and hydrazine. Mono-acylation at 3-amino position of indazole was performed by consecutive diacylation and deacylation reaction. Buchwald-Hartwig palladium catalyzed amination and deprotection provided 3,5-diaminoindazole **6a, b**.

Syntheses of indazole substituted with 4-amino-3-fluorobenzamide derivatives were carried out with 2-fluoro-5-nitrobenzonitrile as shown in Figure 3. Various amines were introduced to 3-fluorobenzoic acid moiety which is at *N*5 position of 3,5-diaminoindazole while keeping 4-ethoxyphenylacetyl group at *N*3 position. After Buchwald-Hartwig palladium catalyzed amination with ethyl 4-bromo-3-fluorobenzoate, derivatized compound was obtained by ester hydrolysis followed by amide coupling. The structures of the synthesized compounds were characterized by ^1^H NMR, ^13^C NMR and ESI-MS [See Additional file 1].

The *in vitro* anti-proliferative activities of the synthesized compounds were evaluated by SRB assay [13] against human cancer cell lines and the results are shown in Table 1.

**Table 1:** Anti-proliferative activity of the synthesized compounds against human cancer cell lines

	**IC**_**50 **_**(μM)**^**a**^			
	**AMC-HN4**	**A549**	**Caki**	**SNU-449**
**Compd B**^**b**^	93%	91%	81%	-
**Adriamycin**^**b**^	89%	52%	65%	-
**5-FU**	>10	4.9 ± 1.5	>10	>10
**6a**	0.37 ± 0.10	1.0 ± 0.1	7.2 ± 1.4	1.7 ± 0.5
**6b**	0.71 ± 0.17	1.3 ± 0.2	>10	3.8 ± 1.0
**9a**	0.21 ± 0.04	1.5 ± 0.2	11.0 ± 0.1	3.1 ± 1.3
**9b**	2.5 ± 0.3	>10	> > 10^c^	>10
**9c**	2.9 ± 0.6	>10	> > 10^c^	> > 10^c^
**9d**	2.6 ± 0.4	>10	> > 10^c^	> > 10^c^
**9e**	>10	> > 10^c^	> > 10^c^	>10
**9f**	>10	> > 10^c^	> > 10^c^	> > 10^c^
**9g**	5.5 ± 1.3	> > 10^c^	> > 10^c^	> > 10^c^
**9h**	2.0 ± 0.5	> > 10^c^	> > 10^c^	>10

^a^Data are mean of three independent experiments ± standard deviation.

^b^% inhibition at 0.75 μM.

^c^No inhibition up to 10 μM.

Modification of substituent at 5-position of indazole was performed based on the previous results that substituent structure at 3-position of indazole influenced on the potency but not the selectivity between cancer cell lines [8]. As a first step, 2-fluoroaniline was introduced instead of 1λ^6^-isothiazolidine-1,1-dione at 5-position of indazole. This approach improved the cell selectivity but resulted in reduction of inhibitory activity, (**B** vs. **6a)**. When 1,1′-biphenyl group was replaced with 4-ethoxyphenyl group, the selectivity over Caki cell was enhanced, (**6a** vs. **6b)**.

A good dependency between the structure and selectivity was obtained by changing the substituent on 2-fluoroaniline. Also, subtle structural differences in carboxamide at 2-fluoroaniline brought a significant change on the growth inhibitory activity. The compound with morpholine **9a** showed high potency on AMC-HN4 with more than 7-fold selectivity over other cancer cells. AMC-HN4 was known less sensitive to 5-FU which is widely used for the treatment of HNSCC, while a little prone to Adriamycin.

Though the activity was dropped by an order of magnitude by switching morpholine to 4-methylpiperazine, the selectivity for AMC-HN4 was not diminished, **(9a** vs. **9b)**. Structurally similar substituents such as 2-(dimethylamino)ethylamine **9c** and 2-(diethylamino)ethylamine **9d** showed similar activity and selectivity while 2-molpholinoethylamine **9e** resulted in drastic loss of activity. The activity difference between 3-(dimethylamino)propylamine **9f**, 3-(diethylamino)propylamine **9g** and 3-(dimethylamino)propyl(methyl)amine **9h** may be understood as the target and its structural information are elucidated. Compounds **6b**, **9a**, and **9b** showed the growth inhibition of other HNSCCs (Table 2). Even though both **9a** and **9b** showed similar potency to AMC-HN3, only **9b** showed high selectivity to AMC-HN3 compared to other cancer cell lines.

**Table 2:** Anti-proliferative activity of the synthesized compounds against HNSCC

	**IC**_**50 **_**(μM)**^**a**^		
	**AMC-HN1**	**AMC-HN3**	**AMC-HN6**
**6b**	0.63 ± 0.04	0.58 ± 0.04	3.6 ± 1.1
**9a**	0.19 ± 0.03	0.23 ± 0.04	2.8 ± 0.9
**9b**	1.3 ± 1.0	0.34 ± 0.12	> > 10^b^

^a^Data are mean of three independent experiments ± standard deviation.

^b^No inhibition up to 10 μM.

The alterations in the function of Epidermal Growth Factor Receptor (EGFR) have been linked to tumor development and progression. Numerous EGFR inhibitors are currently in clinical trials based on the previous studies that EGFR overexpression is detected in 40% ~ 90% of HNSCCs [6]. Phase II trials of gefitinib, selective EGFR tyrosine kinase inhibitor, for recurrent/metastatic HNSCC have shown antineoplastic activity. However, in a phase III study, gefitinib did not improve the response rates or overall survival. The resistance of the EGFR-targeted therapy with gefitinib had been linked with the overexpression of cyclin D1 [14]. It was suggested that the combination of CDK inhibitors with EGFR inhibitors might be a useful therapeutic strategy for HNSCC. Both AMC-HN3 and AMC-HN4 cell have mutations delivering inactivation of p16 and overexpression of cyclin D1 [15]. As a result, the compound showing selective potency to either AMC-HN3 or AMC-HN4 has high potential to show synergistic effect with EGFR inhibitors.

Small molecular drugs that have been used in HNSCC therapy or clinical trial have relatively low cellular potency. For example, 5-FU has IC_50_ > 10 μM (Table 1) while *cis*-platin has IC_50_ values between 2.7 to 36.7 μM [16]. The IC_50_ values of gefitinib are in the range of 0.4 and 14.4 μM [14]. A series of compounds tested in this research displayed comparable AMC-HN4 cellular activity to 5-FU, *cis*-platin and gefitinib. They also have a high level of AMC-HN3 selectivity over other cancer cell lines.

## Conclusions

In summary, we have designed and synthesized a series of *N*3-acyl-*N*5-aryl-3,5-diaminoindazole derivatives, and evaluated their anti-proliferative activity against human cancer cell lines, Caki, A549, AMC-HN1, 3, 4, and 6, and SNU449. The study of structure and activity relationship showed that the selectivity against cell lines could be achieved by modification of substituents at N5-aryl group of 3,5-diaminoindazole. Compound **9a** was the most potent compound with about 7-fold selectivity against cancer cell lines tested. Other compounds such as **9b, c**, **d**, and **h** showed lower potency but increased selectivity. For example, **9b** was very selective for AMC-HN3. It is notable that *N*3-acyl-*N*5-aryl-3,5-diaminoindazole analogues can be used as hits in the development of anticancer drug for HNSCC.

## Competing interests

The authors declare that they have no competing interests.

## Authors’ contributions

JL: Design of target compounds and supervision of the synthetic and pharmacological parts. JK: Design and synthesis of target compounds. VH: collaboration in manuscript preparation. JP: Supervision of biological tests. All authors read and approved the final manuscript.

## Supplementary Material

Additional file 1 Supporting Information. Click here for file
