# Supplementary material for: Synthesis and anti-proliferative activity evaluation of N3-acyl-N5-aryl-3,5-diaminoindazole analogues as anti-head and neck cancer agent
Source: Daru. 2014 Jan 6;22(1):4. doi: 10.1186/2008-2231-22-4 (PMC3896709; doi:10.1186/2008-2231-22-4)
Supplement: Additional file 1 — Supporting Information. [file 2008-2231-22-4-S1.pdf]

Supporting Information

Synthesis and anti-proliferative activity evaluation of *N*3-acyl-*N*5-aryl-3,5-diaminoindazole analogues as anti-head and neck cancer agent

Authors: Jinho Lee*, Jina Kim, Victor Sukbong Hong, Jong-Wook Park

^{1}H NMR and ^{13}C NMR

^1H NMR **6(a)**

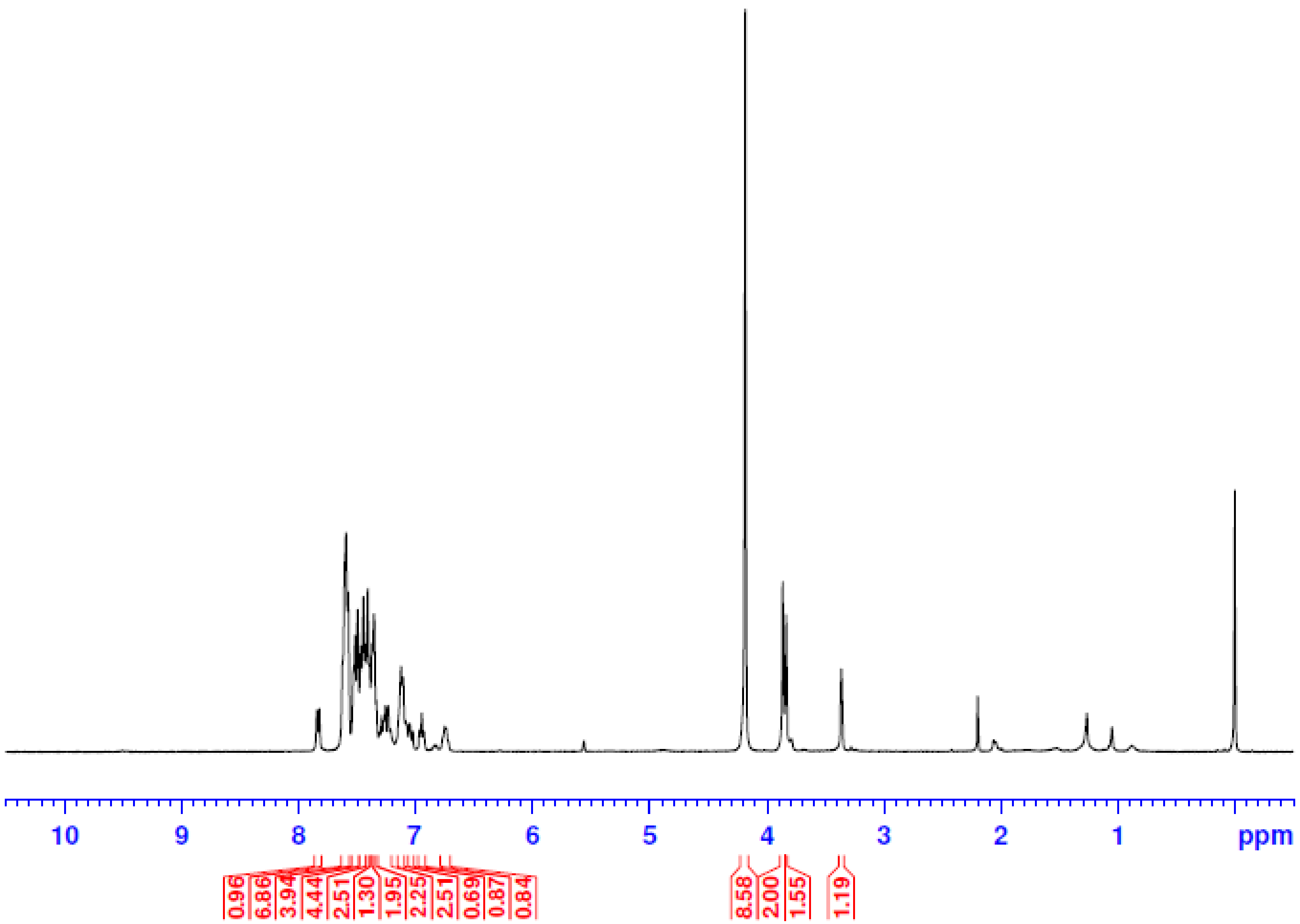
10
9
8
7
6
5
4
3
2
1
ppm
0.96
6.86
3.94
4.44
2.51
1.30
1.95
2.25
2.51
0.69
0.87
0.84
8.58
2.00
1.55
1.19

^{13}C NMR **6(a)**

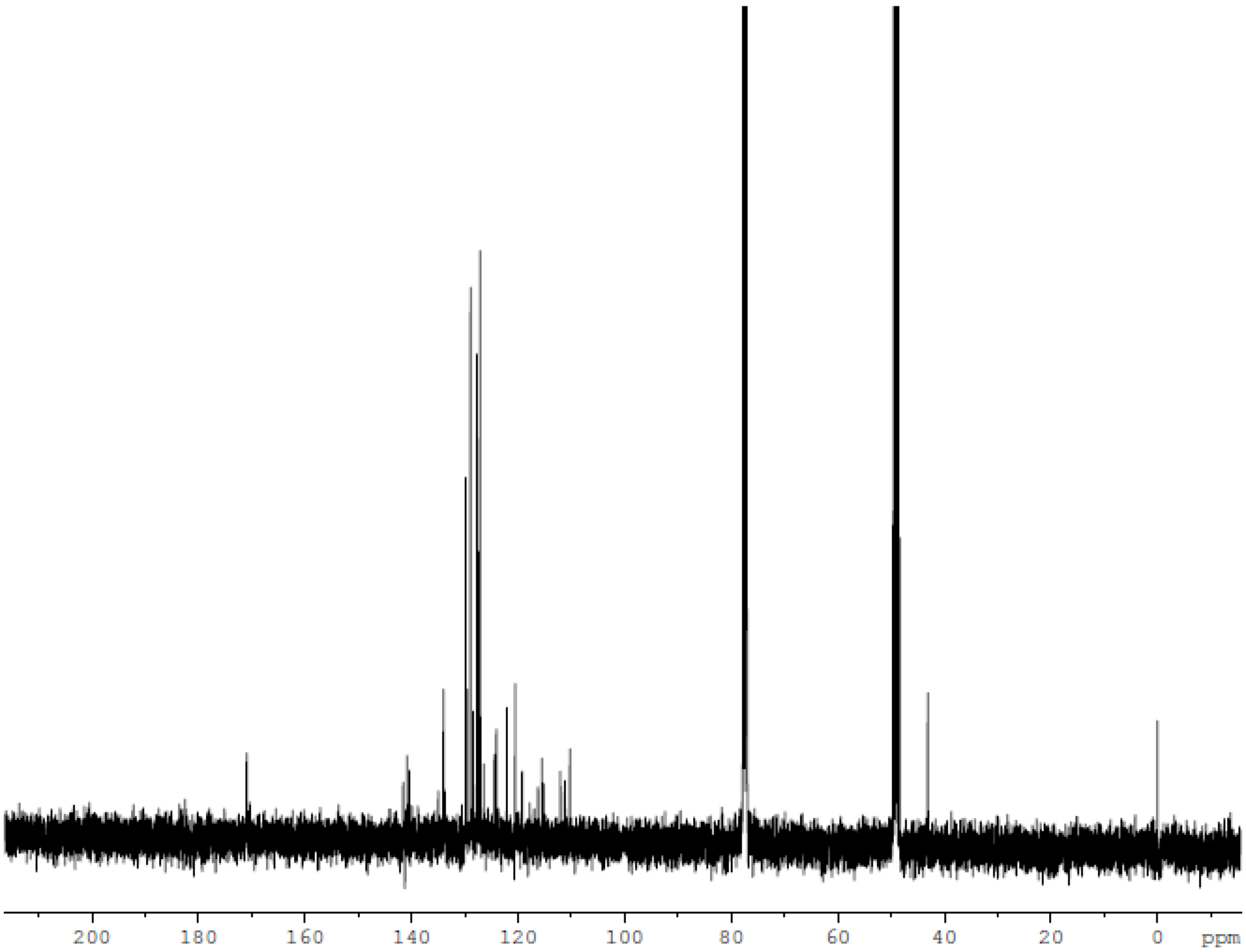

^1H NMR **6(b)**

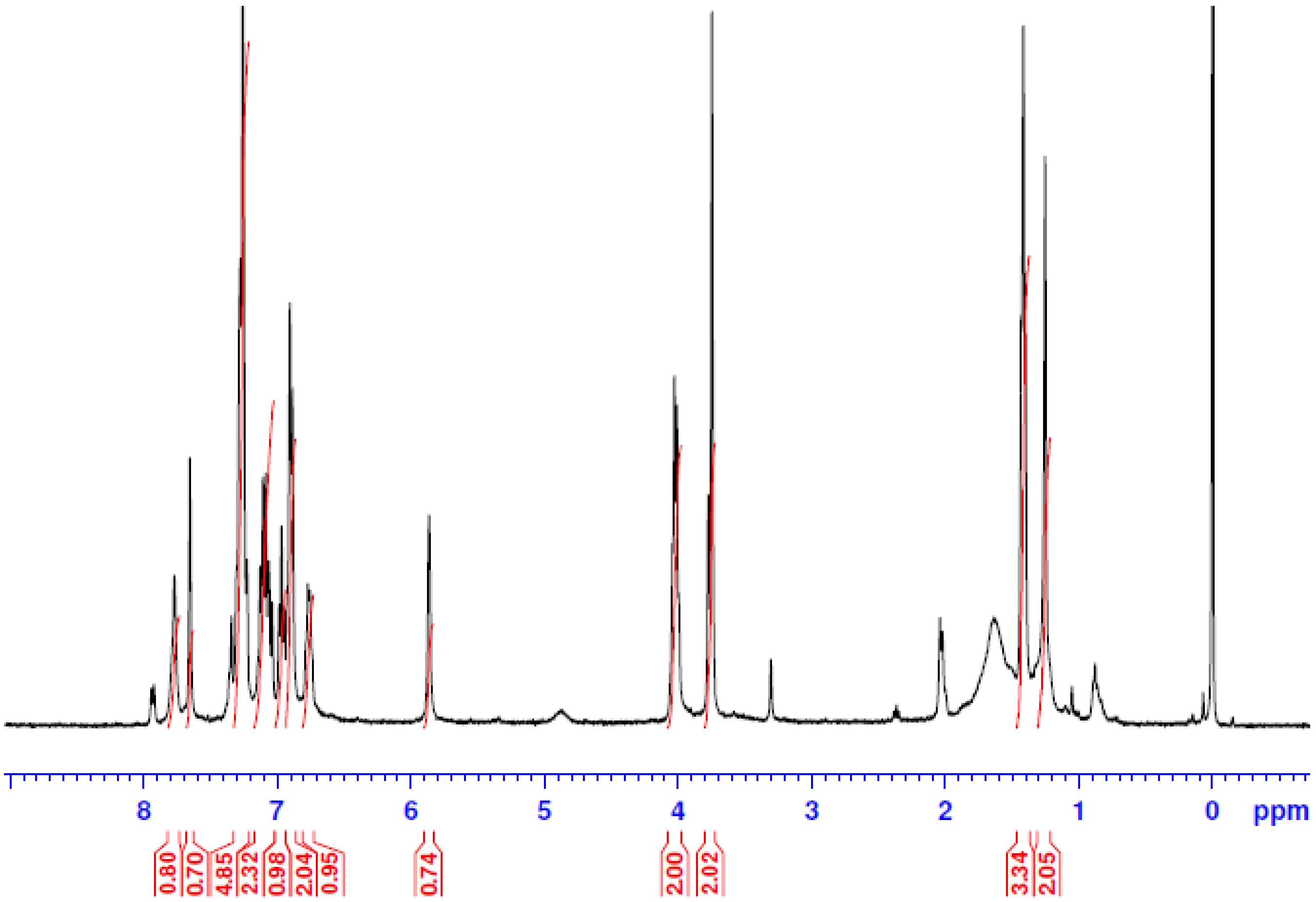

^{13}C NMR **6(b)**

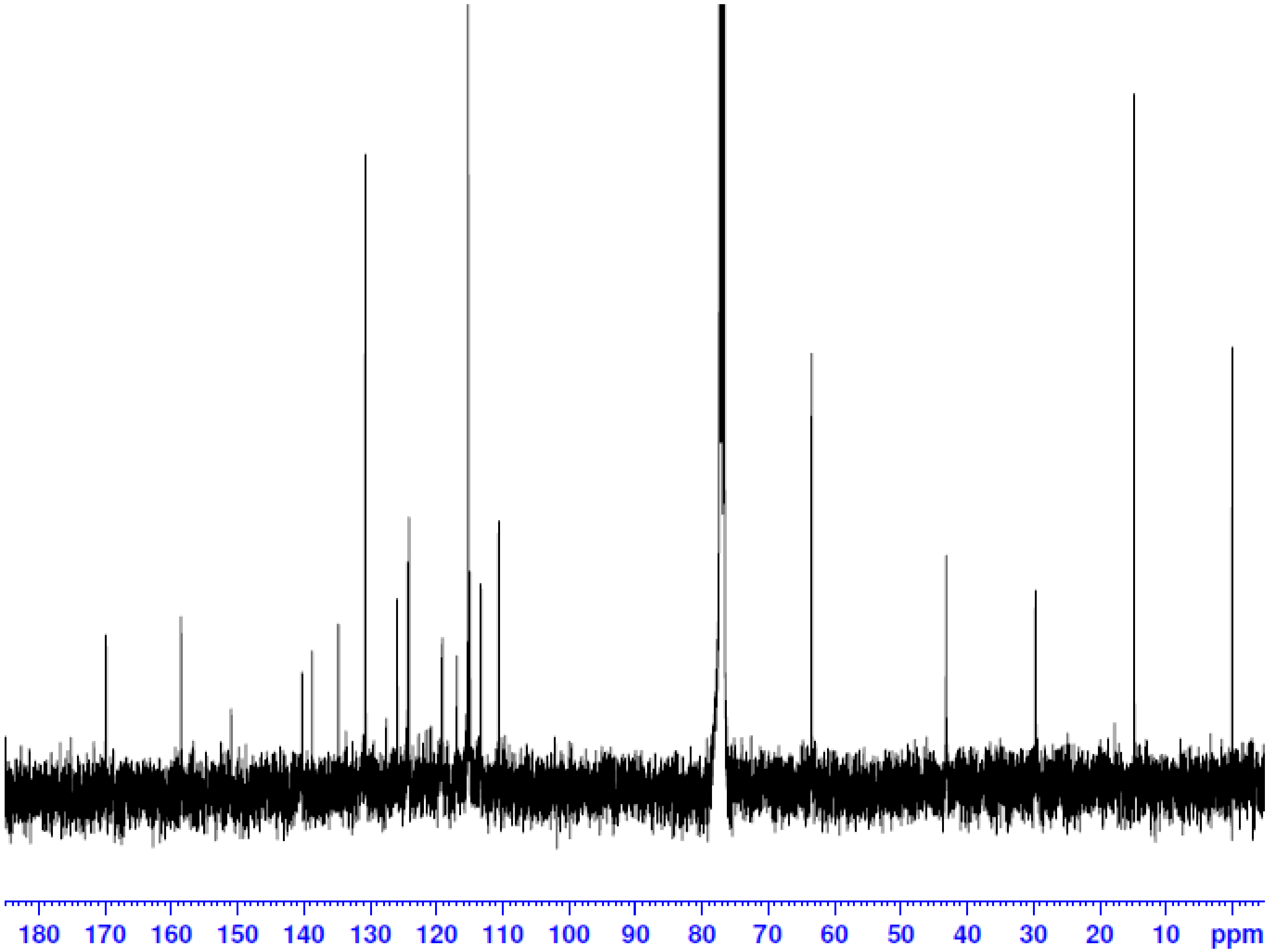

^{1}H NMR **9(a)**

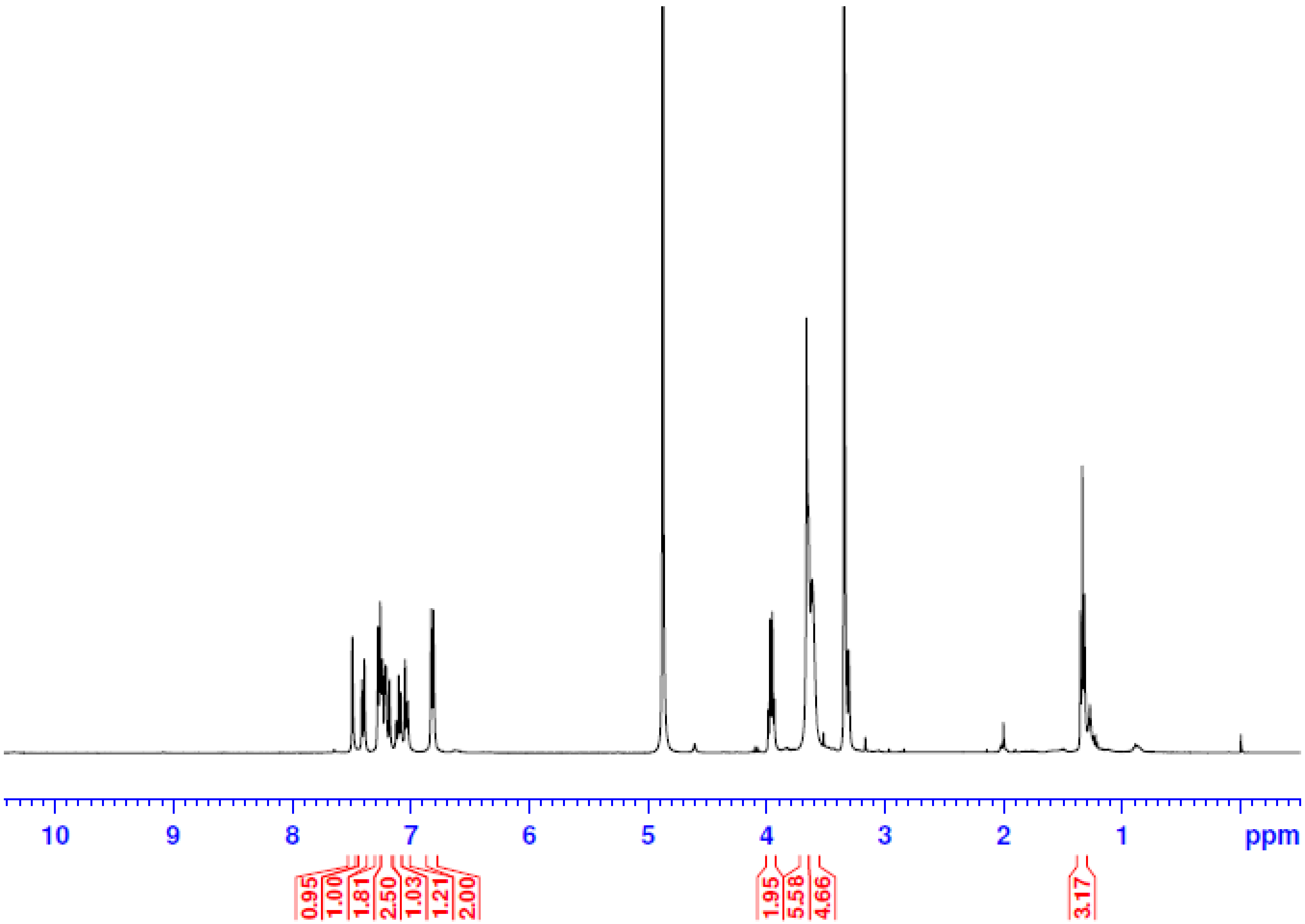

^{13}C NMR **9(a)**

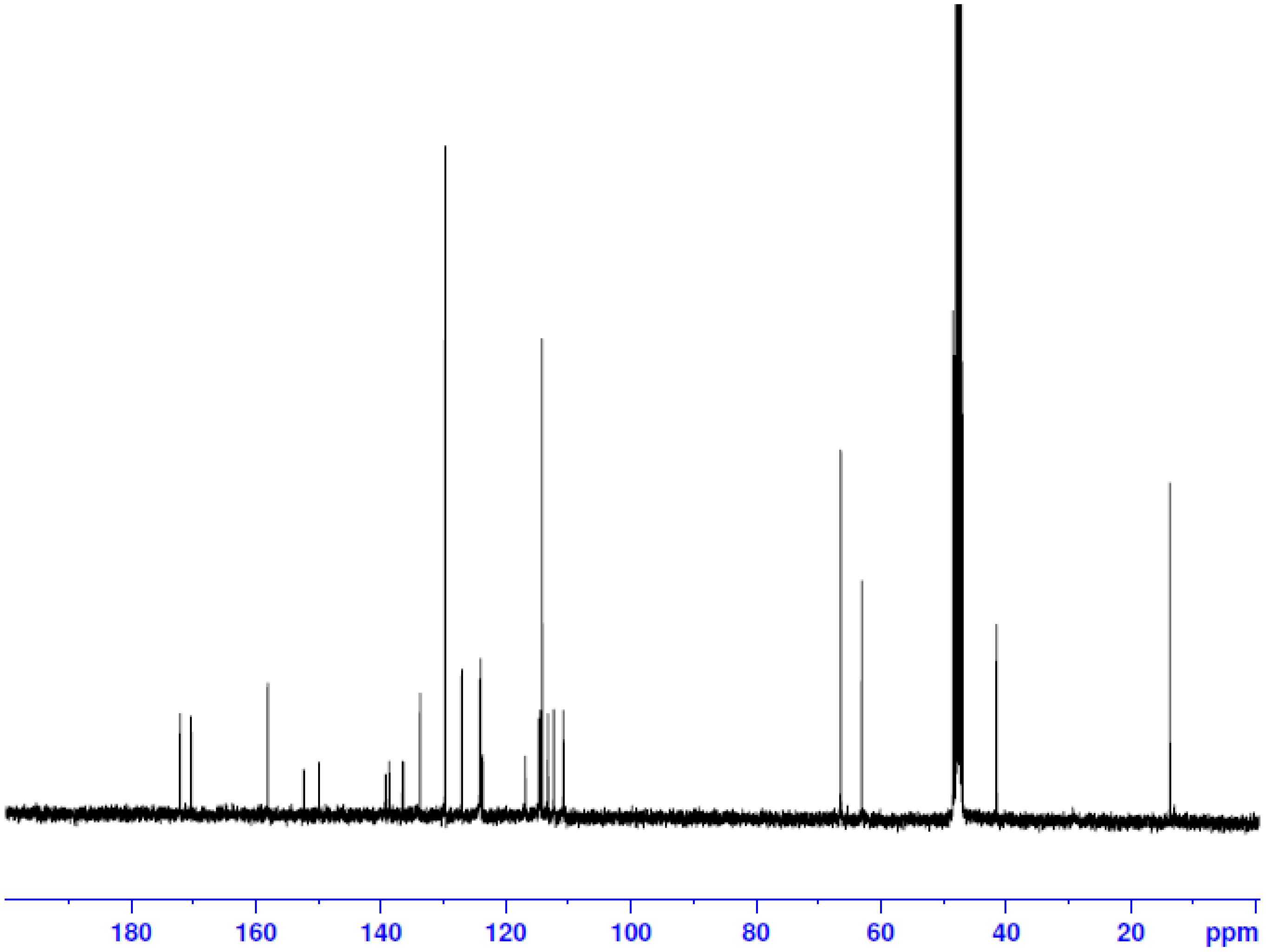

^{1}H NMR **9(b)**

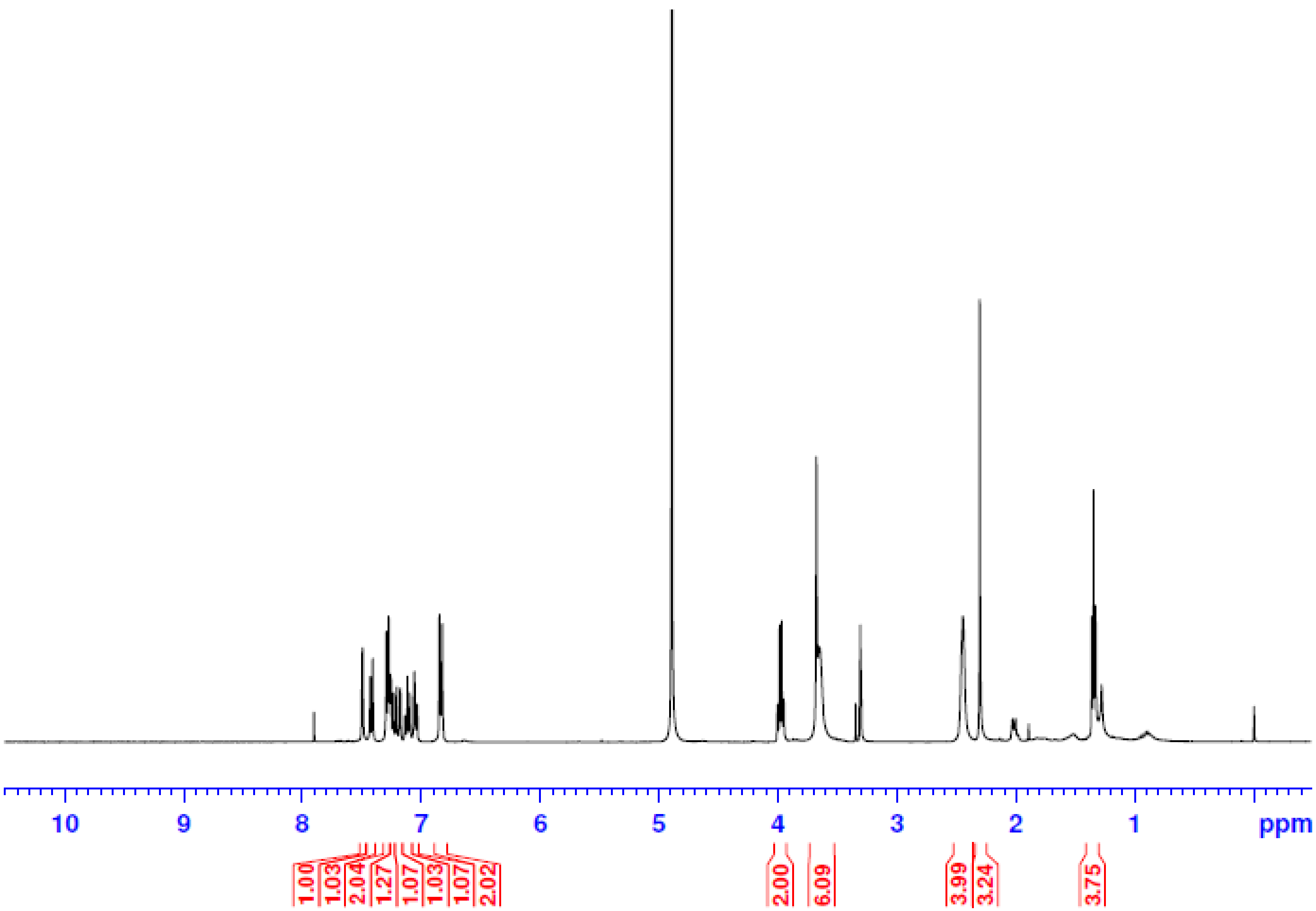
10
9
8
7
6
5
4
3
2
1
ppm
1.00
1.03
2.04
1.27
1.07
1.03
1.07
2.02
2.00
6.09
3.99
3.24
3.75

^{13}C NMR **9(b)**

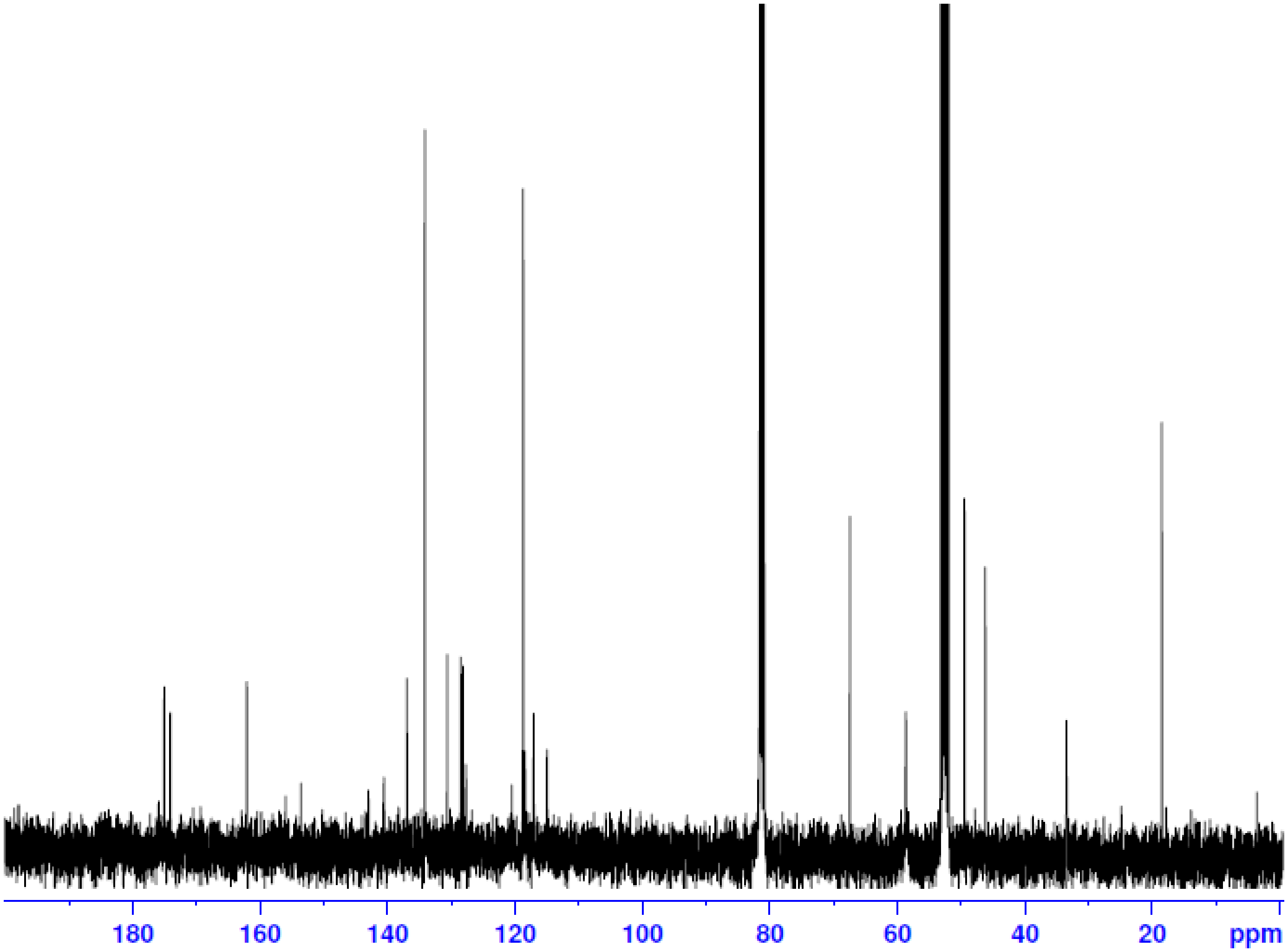

^{1}H NMR **9(c)**

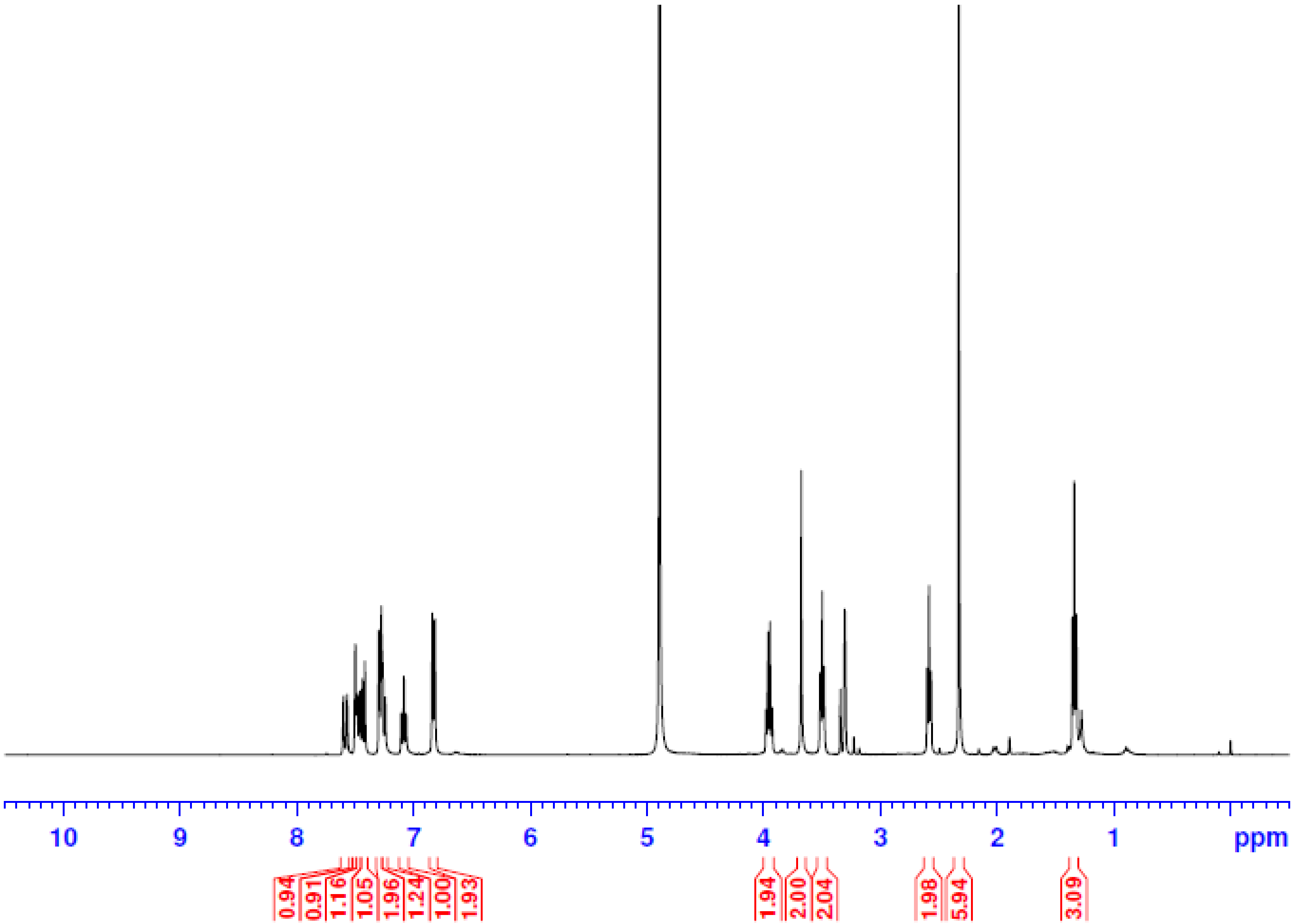
10
9
8
7
6
5
4
3
2
1
ppm
0.94
0.91
1.16
1.05
1.96
1.24
1.00
1.93
1.94
2.00
2.04
1.98
5.94
3.09

^{13}C NMR **9(c)**

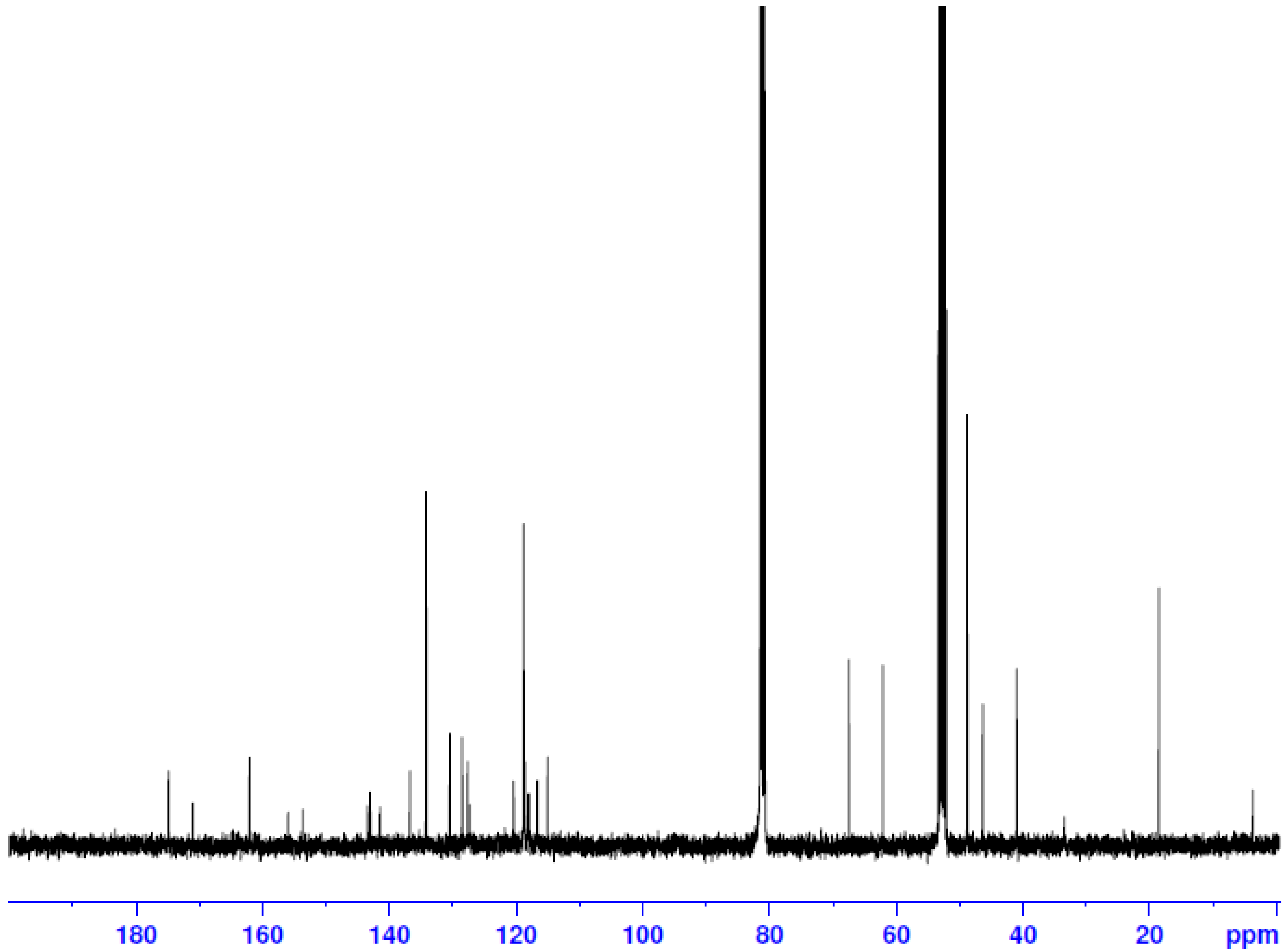

^{1}H NMR **9(d)**

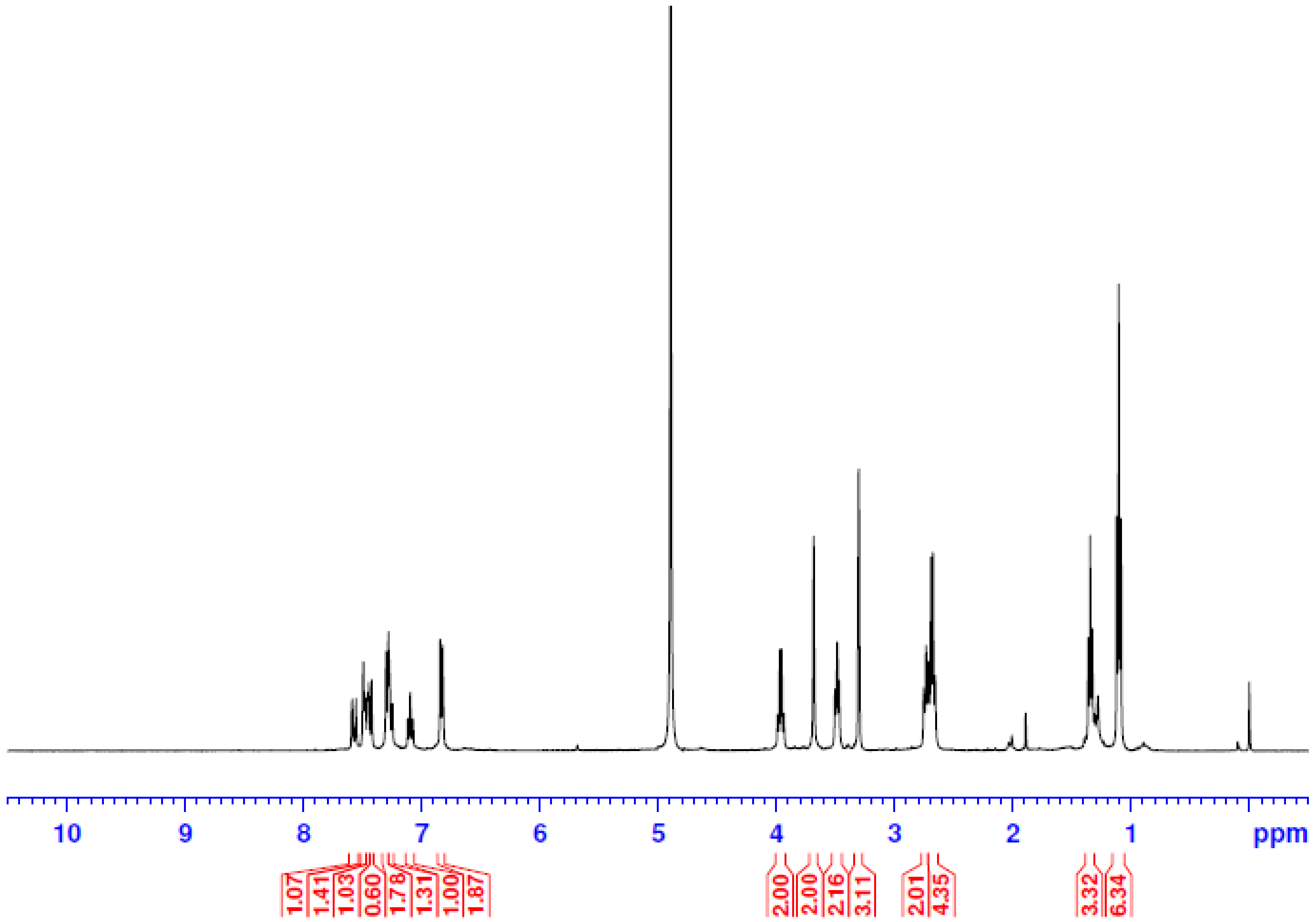

10
9
8
7
6
5
4
3
2
1
ppm
1.07
1.41
1.03
0.60
1.78
1.31
1.00
1.87
2.00
2.00
2.16
3.11
2.01
4.35
3.32
6.34

^{1}H NMR **9(e)**

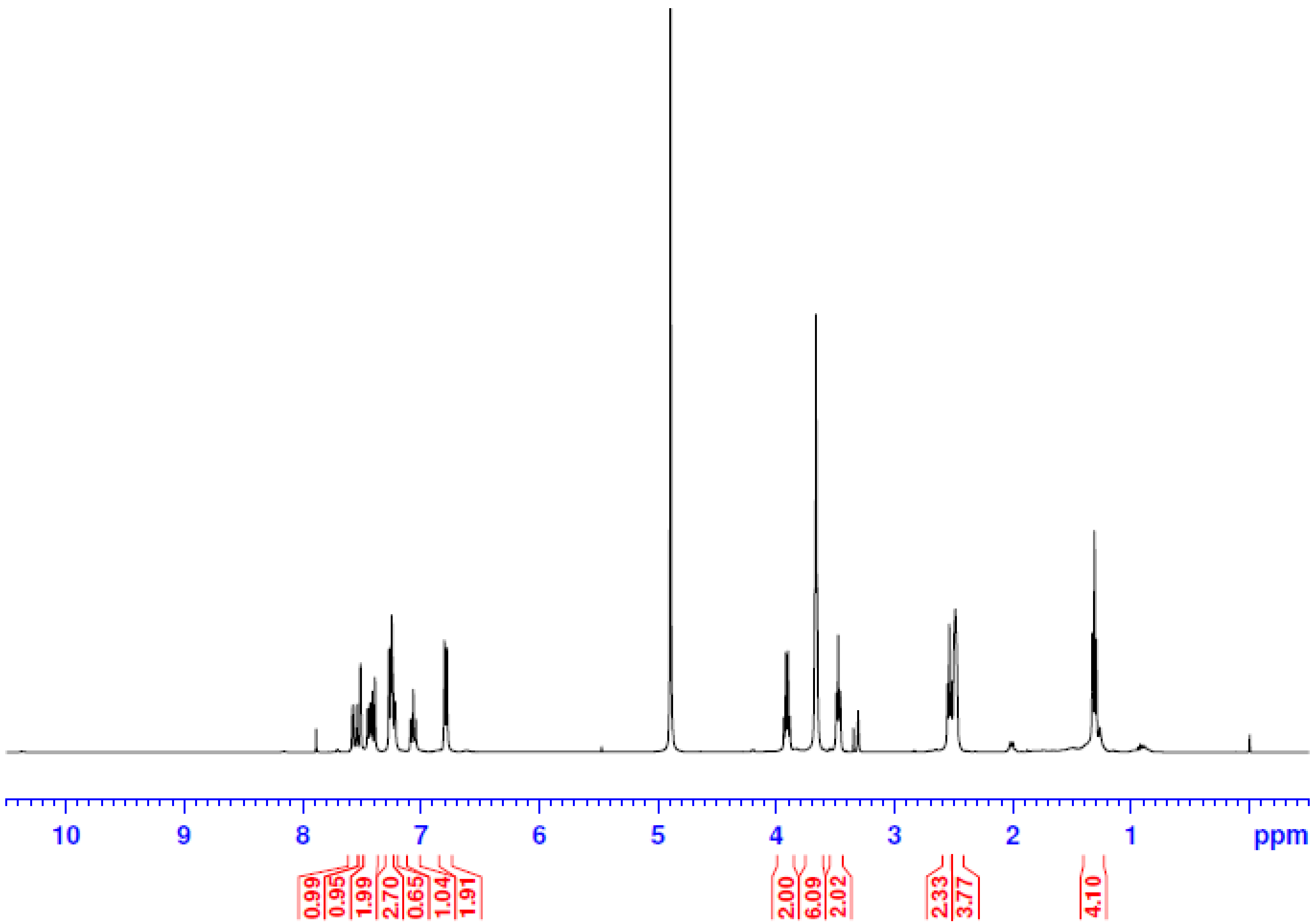
10
9
8
7
6
5
4
3
2
1
ppm
0.99
0.95
1.99
2.70
0.65
1.04
1.91
2.00
6.09
2.02
2.33
3.77
4.10

^{13}C NMR **9(e)**

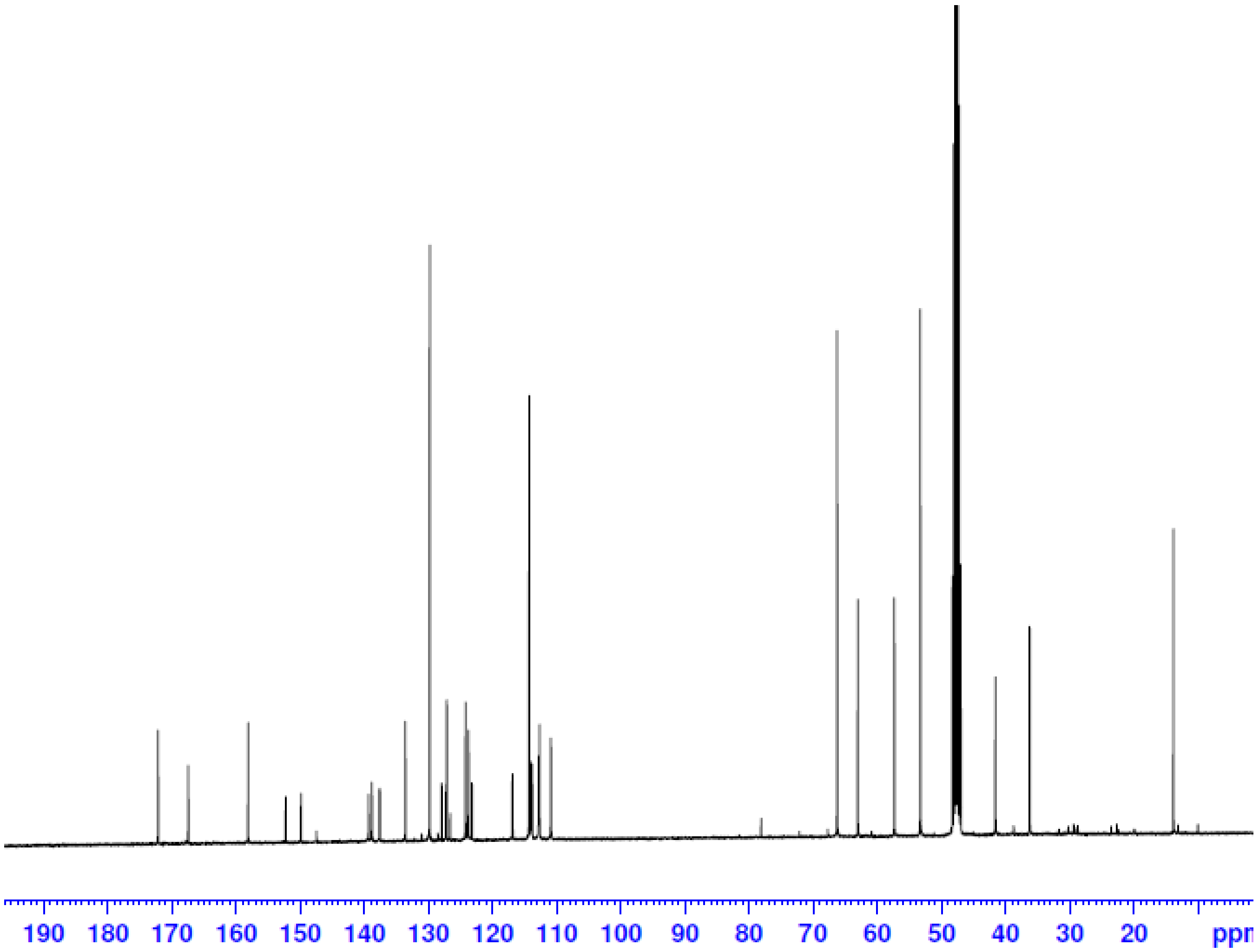

^{1}H NMR **9(f)**

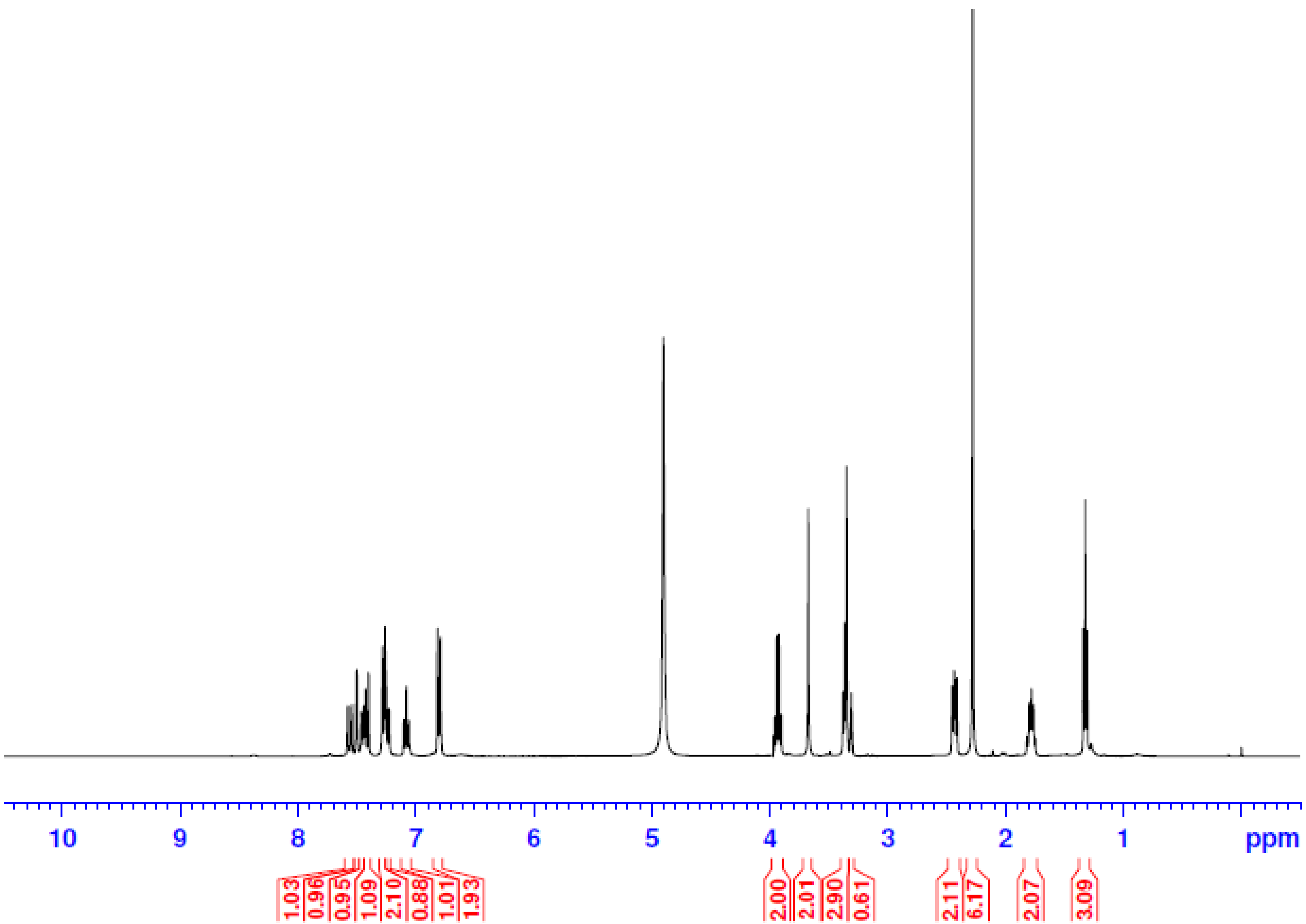
10
9
8
7
6
5
4
3
2
1
ppm
1.03
0.96
0.95
1.09
2.10
0.88
1.01
1.93
2.00
2.01
2.90
0.61
2.11
6.17
2.07
3.09

^{13}C NMR **9(f)**

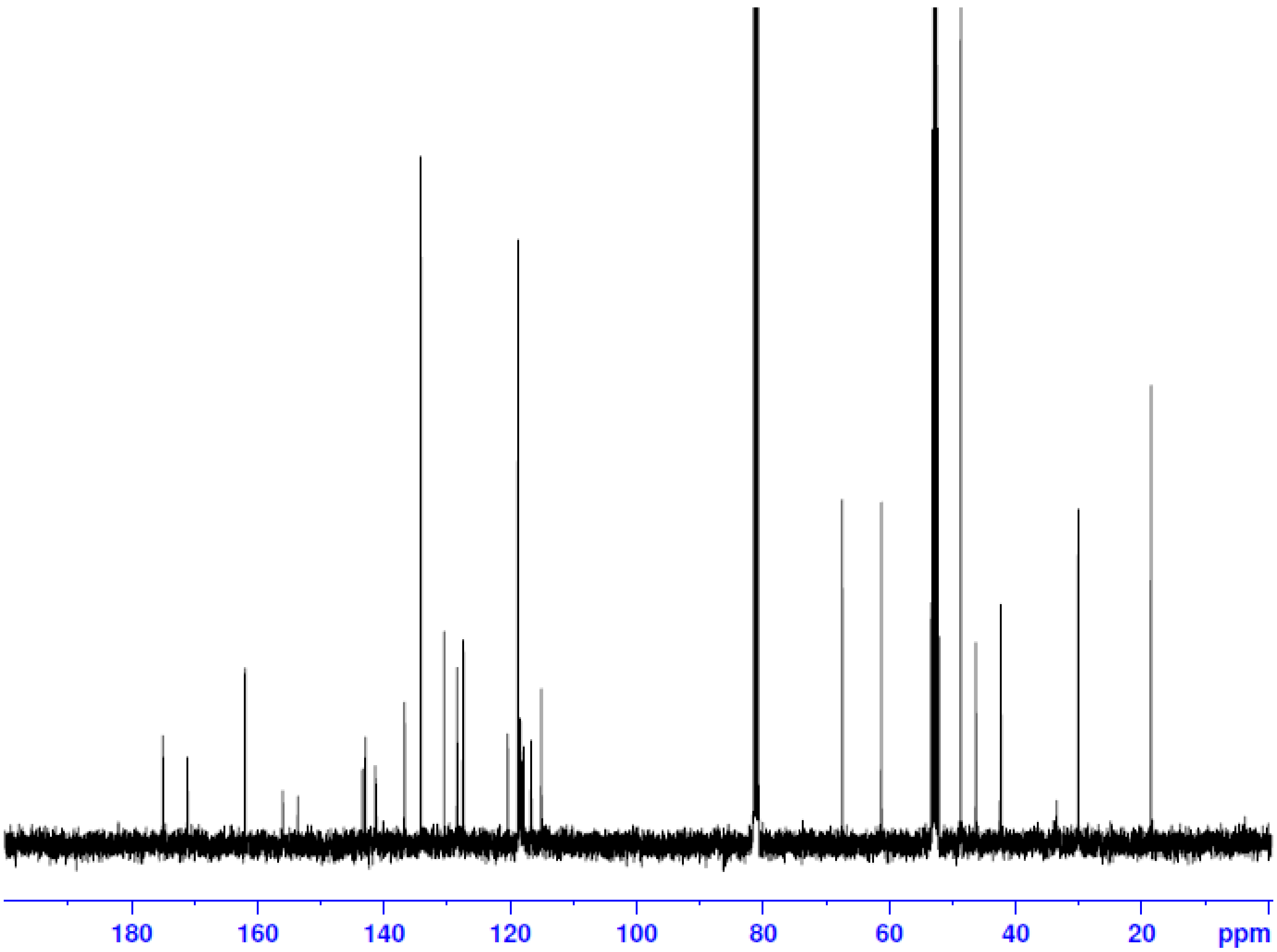

^1H NMR **9(g)**

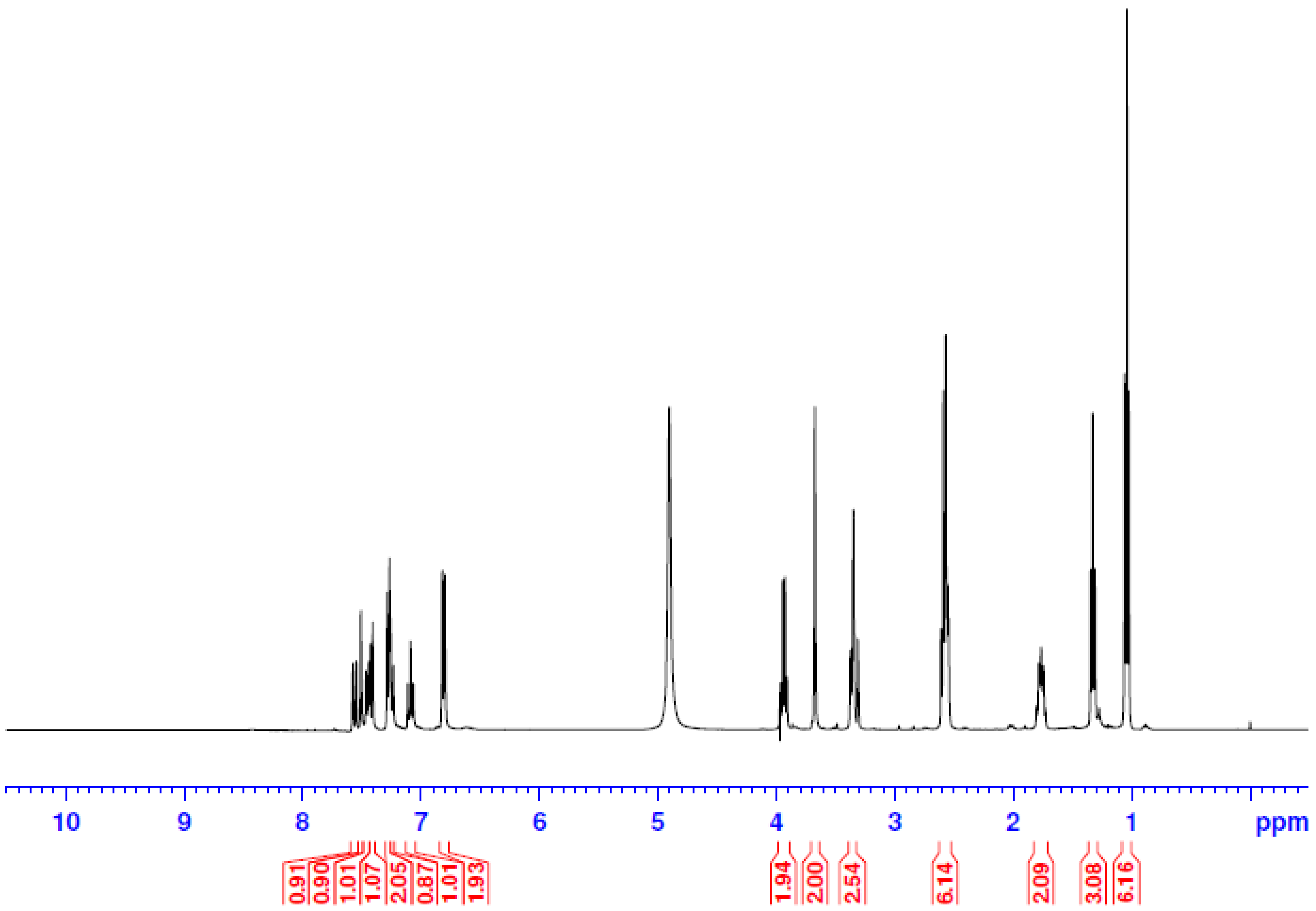

10
9
8
7
6
5
4
3
2
1
ppm
0.91
0.90
1.01
1.07
2.05
0.87
1.01
1.93
1.94
2.00
2.54
6.14
2.09
3.08
6.16

^{13}C NMR **9(g)**

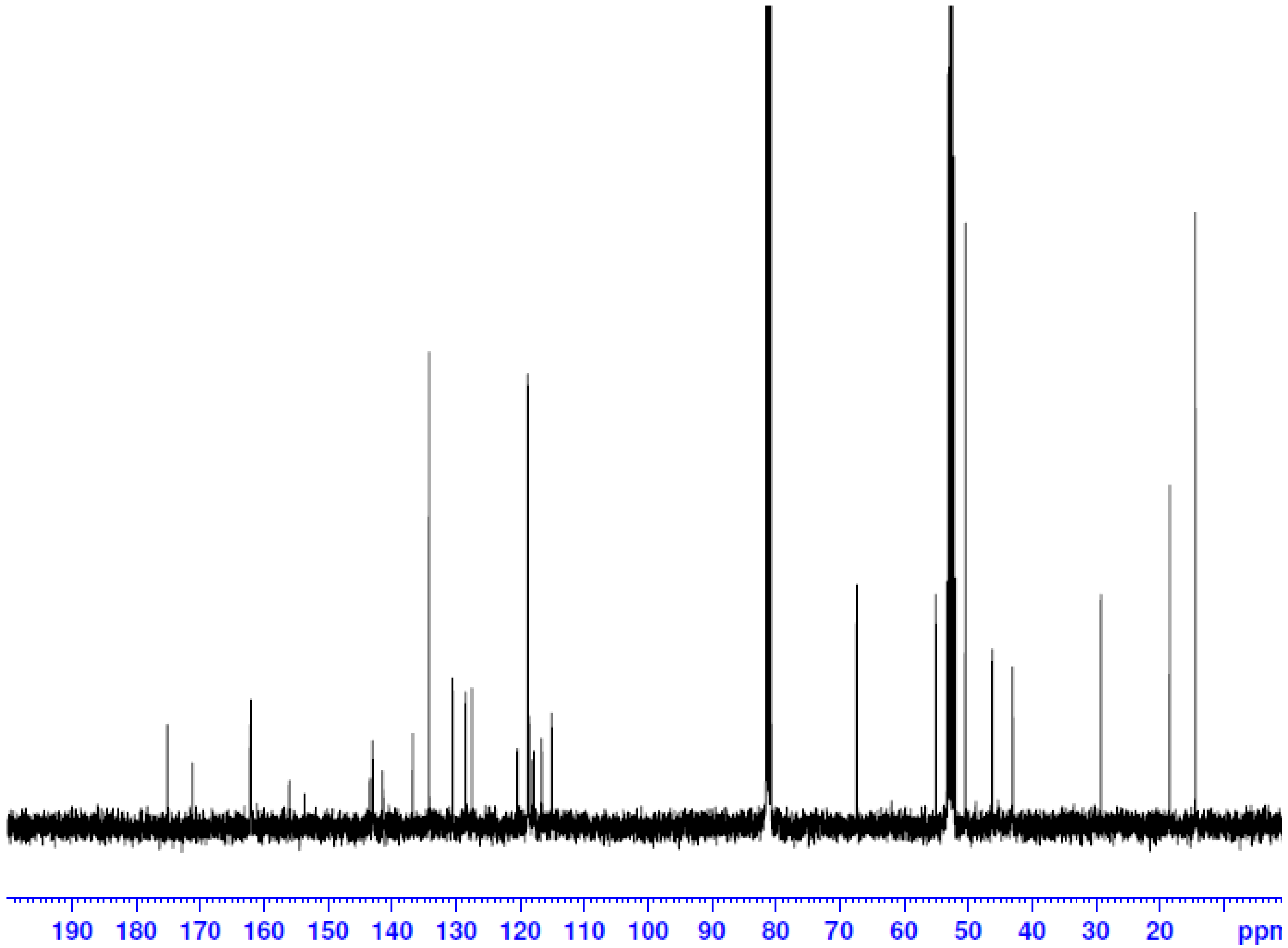

^{1}H NMR **9(h)**

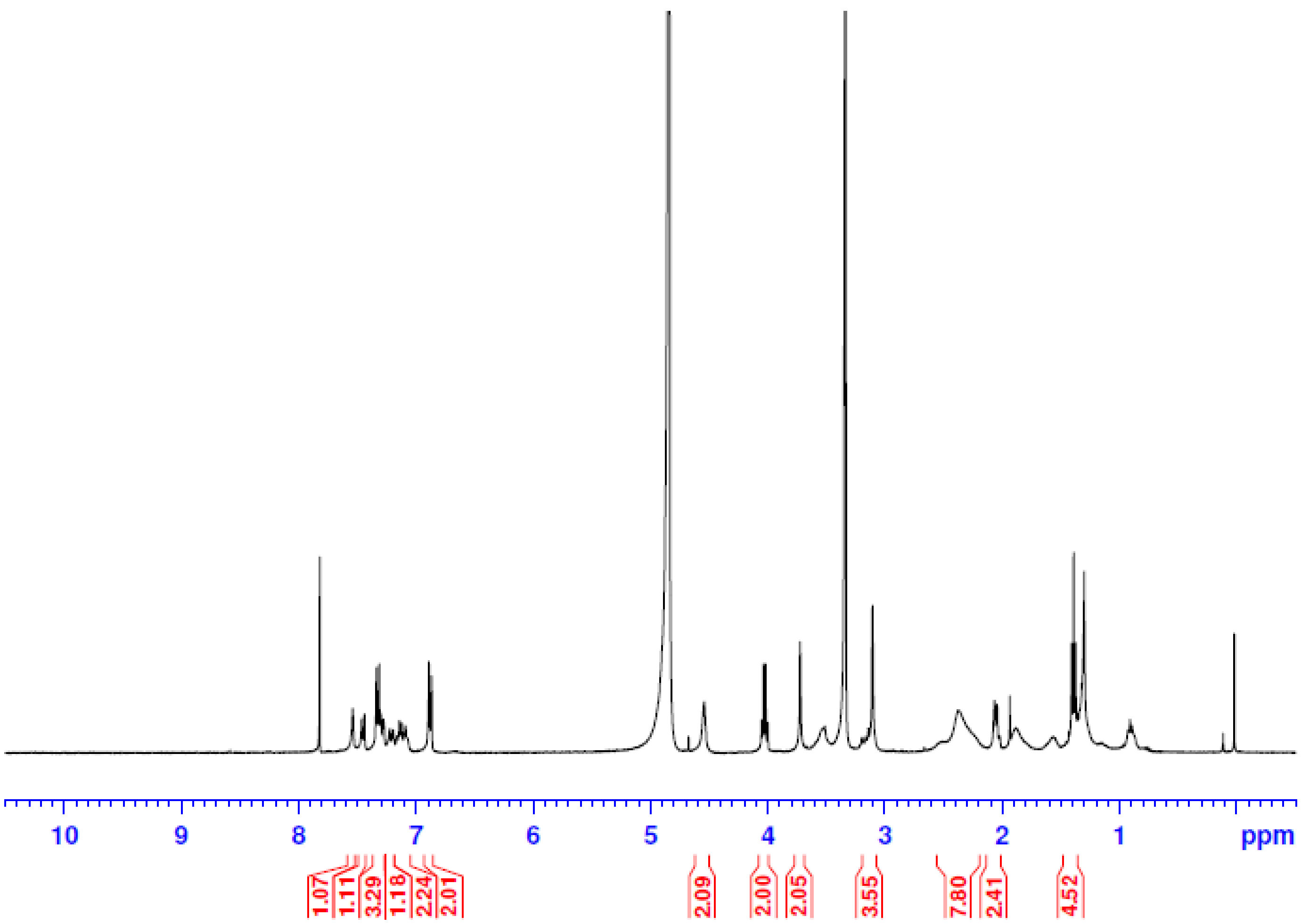
10
9
8
7
6
5
4
3
2
1
ppm
1.07
1.11
3.29
1.18
2.24
2.01
2.09
2.00
2.05
3.55
7.80
2.41
4.52
